# Symplectic encoders for physics-constrained variational dynamics inference

**DOI:** 10.1038/s41598-023-29186-8

**Published:** 2023-02-14

**Authors:** Kiran Bacsa, Zhilu Lai, Wei Liu, Michael Todd, Eleni Chatzi

**Affiliations:** 1 Singapore-ETH Centre, Future Resilient Systems, 138602 Singapore, Singapore; 2 ETH Zurich, Department of Civil, Environmental and Geomatic Engineering, 8093 Zurich, Switzerland; 3 Department of Industrial Systems and Management, NUS, 117576 Singapore, Singapore; 4 HKUST (GZ), Internet of Things Thrust, Guangzhou, 511453 People’s Republic of China; 5 Department of Civil and Environmental Engineering, HKUST, Hong Kong, People’s Republic of China; 6 Department of Structural Engineering, UC San Diego, San Diego, 92093 USA

**Keywords:** Mechanical engineering, Computer science, Applied physics

## Abstract

We propose a new variational autoencoder (VAE) with physical constraints capable of learning the dynamics of Multiple Degree of Freedom (MDOF) dynamic systems. Standard variational autoencoders place greater emphasis on compression than interpretability regarding the learned latent space. We propose a new type of encoder, based on the recently developed Hamiltonian Neural Networks, to impose symplectic constraints on the inferred a posteriori distribution. In addition to delivering robust trajectory predictions under noisy conditions, our model is capable of learning an energy-preserving latent representation of the system. This offers new perspectives for the application of physics-informed neural networks on engineering problems linked to dynamics.

## Introduction

A popular class of methods that find application in system identification and control theory are Gaussian State-Space Models (GSSM), which include the broadly established Bayesian Filters^1,2^. This approach consists in modelling the relationship between the inputs, outputs and inner variables of a dynamic system by a set of differential equations acting on a latent state space; which comprises the so called process model. Noise contamination is assumed both on the process equations, which essentially represent the equations of motion, as well as the measurement equation, which pertains to the observed dynamic outputs. The assumed noise processes are typically modelled as zero-mean Gaussian distributions. GSSMs, such as the Kalman filter^3^, are particularly effective in decomposing a time series into trends and cycles. However, these models require known structures of the differential (process) and algebraic (observation) equations that govern the dynamical system. Such information is often not available a-priori for most complex problems, particularly for those tied to the domain of structural dynamics, where the precise properties (stiffness, restoring force) of the underlying system are often uncertain, or of unknown form^4^. Applying GSSMs for such problems thus requires approximations at the cost of accuracy, often relaxed by resorting to joint state-parameter identification problems, where the model structure is assumed to be defined a priori^5^. Over the years, various data-driven system-identifications techniques have been implemented to correct this lack of prior knowledge so as to extend GSSMs to more complex systems^6–8^.

On a parallel front, new machine learning algorithms, such as the Evidence Lower Bound Optimization (ELBO)^9^ through SGVB (Stochastic Gradient Variational Bayes)^10^, have allowed generative models, such as Variational Autoencoders (VAE), to combine deep learning with stochastic modelling. In recent years, VAEs have been extended to process sequential data for Gaussian processes by introducing a temporal constraint on the dynamics of the latent space^11^. This approach to sequential modelling offers greater flexibility than more conventional state-space models, primarily since VAEs are more apt to learn non-linear dynamics. Models such as Stochastic Recurrent Networks (STORN)^11^ or Deep Markov Models (DMMs)^12^, which are further referred to as DVAEs (Dynamic Variational Autoencoders)^13^, have achieved promising results in speech analysis, music synthesis and medical diagnosis prediction. We note that DVAEs are an unsupervised learning scheme, since the learning of the latent variable is conditioned without directly being connected to the data, in stark contrast with supervised learning methods for dynamical systems such as Nonlinear Autoregressive with Exogenous input models (NARX).

Similar to other deep learning methods, DVAEs do not learn interpretable latent spaces, since they are biased towards learning compressed representations. This poses a problem for multiple objective tasks, common in Robotics, Control, Structural Health Monitoring (SHM) and Prognostic Health Management (PHM) applications, where in addition to adequate response predictions, we seek to also extract meaningful information on the dynamics of the system^14^. To address this issue, we propose to add extra assumptions on the inference network. Our proposed approach adopts a Neural ODE (NODE)^15^ as the a posteriori model for a DVAE; we essentially add the prior information on the distributions being generated via underlying differential equations. Furthermore, we parametrize the integration of our ODE using a symplectic integrator. For a symplectic integrator, the forward integration step splits the phase-space into its displacement and momentum subspaces and updates these subspaces based on Hamiltonian constraints. This imposes an area-preserving property in the phase space on the trajectories of the observed MDOF system. For our systems, we will assume that phase-preservation and energy-conservation properties are equivalent^16^. We thus postulate that our model is able to performed unsupervised statistical learning with energy constraints.

Other recent papers^17,18^ have also explored the idea of symplecticity in time-series analysis. We outline our own contribution as follows:

- We propose a novel framework that combines DMMs with physics-informed machine learning in the form of a NODE. This allows us to explore the use of physics-informed neural networks for statistical unsupervised learning.
- We introduce a symplectic constraint on the learned latent space by using Hamiltonian Neural Networks (HNN) for a posteriori distribution learning.
- We show that our model is able to learn latent quantities, such as energy, in addition to being a state-space observer.
- We study the use of a modified symplectic integrator for cases where the dynamical system’s energy is not constant, such as dissipative and forced systems. We note that we only deal with systems whose Hamiltonian is separable when expressed in Cartesian coordinates.

We note that our the present method does not aim to replace current SHM system identification methods. Rather we focus on demonstrating that deep learning methods can be biased with apriori physical knowledge for an improved performance. Using small linear systems as a starting point, we incrementally prove the efficacy of our method on more and more complex, i.e. higher nonlinearity and DOF, systems.

## Related work

**Figure 1:**
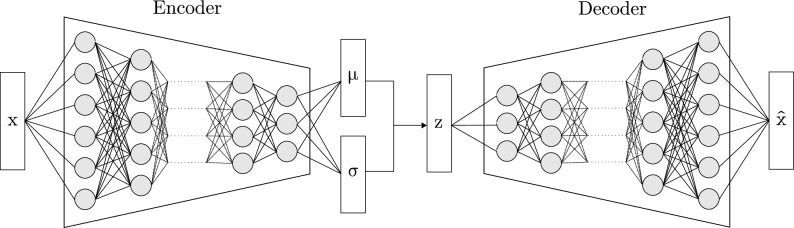
VAE: The data is first passed through an encoder network to output the mean and variance of the latent distribution of the data. Then we sample from this distribution to obtain a candidate latent space. This candidate latent space is then mapped back to an approximation of the original data using a decoder network.

Introduced by Kingma and Welling^9^ and Rezende et al.^10^, the VAE is one of the most popular approaches in stochastic unsupervised learning. The main function of the VAE is to learn a low-rank latent space that encodes noisy observations from a dataset. During the learning phase, the inference of the latent space from the data is referred to as the encoding, in contrast to decoding, which is the generation of future samples from the latent space. Hence the term autoencoder, since the decoder’s distribution should reproduce that of the encoder’s. A summary of the inner workings of the VAE is provided in Fig. 1. VAEs have been extended to noisy time-series autoencoding in a novel group of models referred to as Dynamic Variational Autoencoders (DVAE)^13^. This time-dependency can be modelled in different ways. Bayer and Osendorfer^11^ introduced STORN, where the decoding takes into account the previous states of the model in addition to the current state. Krishnan et al.^19^ included the idea of a Markov property on the latent space in their Deep Kalman filter (DKF). Similarly to a Kalman filter, the latent space, updated during the encoding, is the result of a combination of the VAE inference and its state at the previous step. Chung et al.^20^ combined both these ideas (temporal relationship for both encoding and decoding) in their Variational Recurrent Neural Networks (VRNN). In our work, we will mainly refer to the Deep Markov Model^12^. This model is similar to the DKF, with added gating functions on the latent space update.

Recurrent Neural Networks and their variants, such as Long Short-Term Memory (LSTM) Networks^21^ form the most widespread deep learning algorithm for time-series analysis. Therefore, many of the previously cited DVAEs rely on RNNs as their basic feature extractors. While efficient at finding time-based correlations in the data, it is not possible to add any prior knowledge of the dynamics of the data to an RNN. Chen et al.^15^ introduced the Neural ODE (NODE), a Residual Neural Network (ResNet)^22^ repurposed to learn the flow of the dynamics of an observed system. Each layer of the neural network parametrizes the forward step integration of the underlying differential equation.

Further works in NODEs by Rubanova et al.^23^ have shown that such learning schemes can also be applied to latent variables, i.e., in lifted spaces that have a different dimension from the original observed dataset.

Starting with Raissi et al.^24^, deep learning has also found success in physical modelling. Physics-informed Neural Networks (PINN) have achieved state-of-the-art results in fluid mechanics^25^ and aerodynamics^26^. In the case of structural dynamics, several classical architectures such as CNNs^27^, LSTMs^28,29^, as well as Neural ODEs^30^, have achieved state-of-the-art performance for structure identification and modelling. Wei et al.^31^ have shown that the stochastic nature of VAEs and their subvariants such as the Deep Markov Model^12^ can be used to model uncertain dynamics. Further works on generative models have shown their efficacy when applied to fluid dynamic problems. Zabaras and Geneva^32^ trained a physics-constrained variational model to predict turbulent flows governed by Navier-Stockes equations. Rasul et al.^33^ designed an autoregressive deep learning model, where the data distribution is represented by a conditioned normalizing flow. Their model demonstrate state-of-the-art performance when predicting the dynamics of a flow through a system of pipes.

Hamiltonian Neural Networks (HNN)^34^ are a subset of PINNs that parametrize an explicit formulation of the Hamiltonian of the system being observed. This introduces the notion of energy conservation of the latent space. Saemundsson et al.^35^ opted instead to parametrize the integration of the dynamic system. Such integrators for Hamiltonian systems are known as symplectic integrators. They allow the update of the latent space to be phase-preserving, a notion close to energy conservation^16^.

We note that we are not the first to make the connection between symplectic integration and NODEs. Other works^17,18,36,37^ have already explored the idea of learning symplectic flows with modified NODEs. We note in particular the UniCORNN proposed by Rusch and Mishra, which show that the introduction of Hamiltonian constraints in RNNS increases the learning stability by mitigating the exploding/vanishing gradient problem. This is achieved by computing the second order derivative of each hidden layer of the RNN with symplectic constraints. Our main contributions lies in the incorporation of symplectic flows to the encoder of DVAEs, mainly the DMM. Furthermore, we extend our model to learn the dynamics of non-autonomous systems.

Moreover, we remark that we are not the first to introduce the idea of Hamiltonian dynamics for VAEs. Wolf et al.^38^ showed that Hamiltonian Markov Chain Monte-Carlo (MCMC) can be introduced for the sampling of the posterior. Hamiltonian MCMC is an alternative sampling method to the Metropolis-Hastings algorithm used in the original work on VAEs. In the case of Metropolis-Hastings, samples are accepted or rejected based on a threshold calculated using the target distributions density function (or a proxy function proportional to said density, as it is the case in Variational Inference). In the case of Hamiltonian MCMC, the target distribution is interpreted as a position vector, and an auxiliary momentum distribution is added. A new sample is generated by integrating the position and momentum distributions forward in time along energy-preserving trajectories with a random momentum value. The new sample is accepted or rejected based on the same thresholding scheme of the Metropolis-Hastings algorithm. The added benefit of the Hamiltonian MCMC is that it enables the sampling of distant states that are still highly probable due to the energy-preserving properties of the Hamiltonian integration scheme. This reduces the correlation between two subsequent samples and allows for a better global convergence of the sampling algorithm. Wolf et al.^38^ showed that using Hamiltonian MCMC improved the global convergence for the approximation of the posterior distribution. Caterini et al.^39^ mention that Wolf et al.’s estimation method is not amenable to the reparameterization trick, an essential step towards training neural networks for variational inference. Instead, they use Hamiltonian Importance Sampling^40^, an annealed version of Hamiltonian MCMC to enforce compatibility with the training of a Hamiltonian VAE. Wang and Delinguette^41^ introduced Quasi-symplectic Langevin VAEs, a similar model to the Hamiltonian VAE that replaces the Hamiltonian dynamics with Langevin dynamics according to Langevin’s stochastic differential equation which describes the motion of a particle in a fluid.

## Methodology

### Deep Markov model

#### The variational autoencoder (VAE)

As previously mentioned, the VAE is a stochastic version of an autoencoder. An autoencoder is a DNN trained to match the predicted output to its original input ($\textbf{x} \approx \hat{\textbf{x}}$) for $\textbf{x} \in \mathbb {R}^F$. The autoencoder is parametrized by two neural networks. First, the encoder DNN maps the input vector $\textbf{x}$ to its latent representation $\textbf{z}$ with $\textbf{z} \in \mathbb {R}^G$ and $G \le F$. The decoder DNN then inverts the previous transformation by mapping the latent variable $\textbf{z}$ back to the original input $\textbf{x}$. The VAE^9^ extends the concept of an autoencoder to stochastic latent variables. The encoder is used to infer the Probability Density Function (PDF) of the latent variable $\textbf{z}$ for the data vector $\textbf{x}$. Within the context of Bayesian inference, the PDF of the decoder for $\textbf{x}$ is:

1 $$\begin{aligned} p(\textbf{x}, \textbf{z}) = p_{\theta }(\textbf{x} \vert \textbf{z}) p_{\theta }(\textbf{z}) \end{aligned}$$

with $\theta$ the parameters of the generative model, and where $p_{\theta }(\textbf{z})$ is the prior distribution of the latent variable. In most cases it is assumed to be a unit Gaussian, i.e. $\textbf{z} \sim \mathcal {N}(\textbf{0}_L,\,\textbf{I}_L)$.

This latent variable is sampled from its inferred PDF and then passed through the decoder to generate a new datapoint $\hat{\textbf{x}}$. For the VAE, the PDF of $\textbf{z}$ is assumed to be Gaussian. Th assumption hold for most applications of the VAE. However, for datasets with non-gaussian distributions, the latent space can be deformed a non-gaussian distribution using Neural Autoregressive Flows^42^, i.e., a series of invertable, smooth and trainable transformations.

#### DVAE and ELBO

Our DVAE model reuses the DMM structure from Krishnan et al.^12^. The choice of this model is motivated by the unsupervised training for the discovery of the latent space that encodes the temporal dynamics of the data. In addition, the latent space of the DMM has an enforced Markov property, i.e. the model assumes that the current state of the latent space can be inferred from its previous state; an assumptions that holds true for linear mechanical systems. In the remainder of the paper, we will adhere to the formalism from Girin et al.^13^ so that our proposed model can be easily compared to other DVAEs.

**Figure 2:**
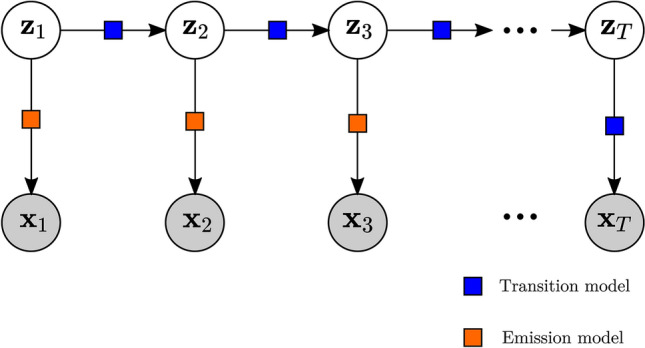
Illustration of the DMM generative process. The latent random variable $\textbf{z}_t$ a function of its previous instance $\textbf{z}_{t-1}$ passed through the transmission model, enforcing a Markov property on the latent variable. At each time step, observations $\textbf{x}_t$ are generated by the emission model.

We consider a sequence of *T* observed random vectors of dimension *F* such as $\textbf{x}_{1:T} = \{\textbf{x}_t \in \mathbb {R}^F \}_{t=1}^{T}$ to which we associate a sequence of latent random vectors of dimension *G* such that $\textbf{z}_{1:T} = \{\textbf{z}_t \in \mathbb {R}^G \}_{t=1}^{T}$. Both $\textbf{x}_{1:T}$ and $\textbf{z}_{1:T}$ are stochastic variables. The generative model of the DMM models the probability distributions of both these stochastic variables as follows:

2 $$\begin{aligned}{} & {} \textbf{z}_t \sim \mathcal {N}\left(V_{\beta }(\textbf{z}_{t-1}), S_{\delta }(\textbf{z}_{t-1})\right) \end{aligned}$$

3 $$\begin{aligned}{} & {} \quad \textbf{x}_t \sim \mathcal {N}\left(W_{\eta }(\textbf{z}_t), R_{\kappa }(\textbf{z}_t)\right) \end{aligned}$$

where *V* and *W* are the respective mean models for the latent and observed variables, *S* and *R* are the respective variance models, and $\{\beta , \ \delta \}$, $\{\eta , \ \kappa \}$ are the parameter sets for each model, respectively. From now on, we will group $\beta$, $\delta$, $\eta$, $\kappa$ as the parameter set $\theta$, describing the latent distribution model. Unlike conventional VAEs, we introduce a “Markov property”, i.e. time dependency on the latent variable $\textbf{z}_t$. Furthermore, our model differs from that adopted by Krishnan et al.^19^ in two ways. First we consider the PDF of the observables to be a Gaussian instead of Bernoulli distribution. We also remove the input dependency and only consider the systems from an output perspective. Our model is mainly applicable where the input of the system is unknown or difficult to measure. The generative model is shown on Fig. 2.

As per Kingma and Welling^9^, the true posterior $p_{\theta }(\textbf{z} \vert \textbf{x}) = \frac{p_\theta (\textbf{z})}{p_\theta (\textbf{x})} p_{\theta }(\textbf{x} \vert \textbf{z})$ is intractable. Therefore, common optimization methods, such as expectation maximization cannot be used. The workaround by Kingma and Welling^9^ can be summarized by approximating the posterior distribution with an auxiliary distribution $q_{\phi }$ and optimizing the lower bound for the marginal likelihood, such as:

4 $$\begin{aligned} \text {log} \ p_{\theta }(\textbf{x}) \ge \mathbb {E}_{q_{\phi }(\textbf{z} \vert \textbf{x})} \left[\text {log} \ p_{\theta }(\textbf{x} \vert \textbf{z})\right] - D_{\text {KL}} (q_{\phi }(\textbf{z} \vert \textbf{x}) \ || \ p_{\theta }(\textbf{z}) \end{aligned}$$

Where $D_{\text {KL}}$ is the Kullback-Leibner divergence. Both $p_{\theta }$ and $q_{\phi }$ can be parametrized by neural networks that can be optimized using Monte-Carlo estimates to compute the unbiased gradients of $\mathbb {E}_{q_{\phi }(\textbf{z} \vert \textbf{x})}[\text {log} \ p_{\theta }(\textbf{x} \vert \textbf{z})]$. This process is known as Evidence Lower Bound Optimization (ELBO).

#### Training loss

Krishan et al.^12^ point out that, in the case of the inference model, the Markov property implies that all past information at time *t* is contained within $\textbf{z}_{t-1}$. The posterior can thus be factorized as:

5 $$\begin{aligned} p_{\theta } = p_{\theta }(\textbf{z}_1 \vert \textbf{x}_{1:T}) \prod _{t=2}^{T} p_{\theta }(\textbf{z}_t \vert \textbf{z}_{t-1}, \textbf{x}_{t:T}) \end{aligned}$$

This allows us modify the inference model as:

6 $$\begin{aligned} q_{\phi }(\textbf{z}, \textbf{x}_{1:T}) = q_{\phi }(\textbf{z}_1 \vert \textbf{x}_{t:T}) \prod _{t=2}^{T} q_{\phi }(\textbf{z}_t \vert \textbf{z}_{t-1}, \textbf{x}_{t:T}) \end{aligned}$$

Finally, the loss used to optimize both the generative and inference model during the training phase follows from (4) and is given by:

7 $$\begin{aligned} \mathfrak {J}(\theta , \phi , \textbf{x}_{1:T}) =&\sum _{t=1}^{T} \mathbb {E}_{q_{\phi }(\textbf{z}_t \vert \textbf{x}_{1:T})} \left[\text {log} \ p_{\theta }(\textbf{x}_t \vert \textbf{z}_t)\right] - \nonumber \\&\sum _{t=1}^{T} \mathbb {E}_{q_{\phi }(\textbf{z}_{t-1} \vert \textbf{x}_{1:T})} \left[D_{\text {KL}}(q_{\phi }(\textbf{z}_t \vert \textbf{z}_{t-1}, \textbf{x}_{t:T}) \ \vert \vert \ p_{\theta }(\textbf{z}_t \vert \textbf{z}_{t-1}))\right] \end{aligned}$$

**Figure 3:**
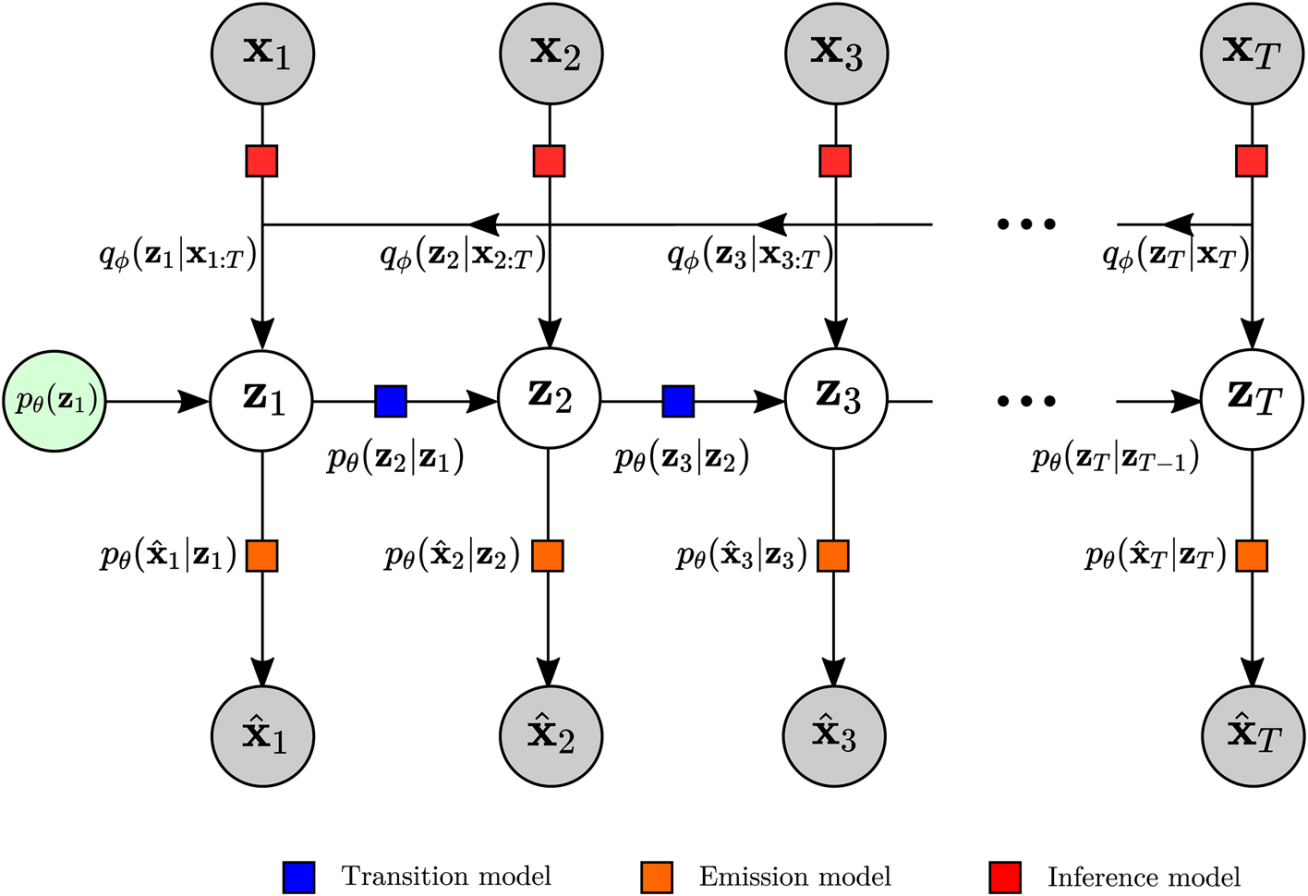
Deep Markov Model: Similarly to the VAE, the data is mapped to a latent representation of the data via the encoder. However, the final latent state is estimated as a weighted average between it’s previous and inferred state. For the first estimated state, the weighted average is computed with a learned prior distribution. This resulting latent state $\textbf{z}_t$ is the one that is passed on to the decoder transition and emission models for an estimation of the data and the subsequent latent state.

The first term of the loss maximizes the likelihood $p_{\theta } (\textbf{x}_t | \textbf{z}_t)$, i.e., it enforces reconstruction accuracy by maximizing the likelyhood that the training data can be generated by the latent variable of the model. The second term is referred to as the information gain^43^. This is because the Kullback-Leibner divergence allows us to minimize the difference between the approximate posterior distribution $q_{\phi }(\textbf{z}_t \vert \textbf{z}_{t-1}, \textbf{x}_{t:T})$ and the Markov transmission prior distribution $p_{\theta }(\textbf{z}_t \vert \textbf{z}_{t-1})$ and acts as a regularizer^9^. Kingma and Welling^9^ have shown that the joint learning of these two terms is equivalent to minizing the Kullback-Leibner divergence between the true and the approximate posterior distributions. The overall computation flow of the DMM is summarized in Fig. 3.

The computation of the ELBO of the DMM requires the direct sampling of $q_\phi (\textbf{z}_t \vert \textbf{z}_{t-1}, \textbf{x}_{t:T})$ to build the expectation. However, the posterior factorization given by Krishnan et al.^12^ in (5) and (6) allows us to employ the following “cascade trick” detailed in Girin et al.^13^ to approximate the intractable expectations in the loss function. The first expectation in this Variational Lower Bound expression can be developed as follows:

8 $$\begin{aligned} \mathbb {E}_{q_{\phi }(\textbf{z}_{t} \vert \textbf{x}_{1:T})}\left[ f(\textbf{z}_t)\right] = \mathbb {E}_{q_{\phi }(\textbf{z}_{1:t} \vert \textbf{x}_{1:T})}\left[ f(\textbf{z}_t)\right] = \mathbb {E}_{q_{\phi }(\textbf{z}_1 \vert \textbf{x}_{1:T})}\left[ \mathbb {E}_{q_{\phi }(\textbf{z}_2 \vert \textbf{z}_1, \textbf{x}_{2:T})}[\dots [\mathbb {E}_{q_{\phi }(\textbf{z}_t \vert \textbf{z}_{t-1}, \textbf{x}_{t:T})}[f(\textbf{z}_t)]]]\right] \end{aligned}$$

where $f(\textbf{z}_t)$ denotes an arbitrary function of $\textbf{z}_t$. The second expectation of the Variational Lower bound can be developed by a similar procedure. Then each intractable expectation can be approximated using Monte Carlo estimates. This requires the sampling of $q_{\phi }(\textbf{z}_\tau | \textbf{z}_{\tau -1}, \textbf{x}_{\tau :T})$, iteratively for $\tau = 1 \ \textrm{to} \ t$ using the same reparametrization trick as in standard VAEs. Thus, the Variational Lower Bound becomes differentiable and can be optimized with gradient-descent-based techniques.

### Neural ODEs

Neural ODEs are a new type of DNN architecture designed for time-series analysis. Under the assumption that the dynamics of a system follows a set of differential ordinary equations, the model attempts to approximate the flow of the system.^15^ reuse the existing ResNet^22^ architecture, where each neural network layer parametrizes a forward flow step.

This is equivalent to approximating a forward integration of the governing ODE using a single layer perceptron. For an arbitrary hidden step $\textbf{h}_t$ at time *t* of the encoding process, for a nondescript forward time step discretization $\tau$, the forward time step is updated with:

9 $$\begin{aligned} \textbf{h}_{t+\tau } = \textbf{h}_{t} + \frac{\text {d} \textbf{h}_t}{\text {d}\tau } \end{aligned}$$

With the approximation using the parameter set $\psi$ as the weights of our neural network :

10 $$\begin{aligned} \frac{\text {d} \textbf{h}_t}{\text {d}\tau } = \Phi (\textbf{h}_t, \tau , \phi ) \end{aligned}$$

**Figure 4:**
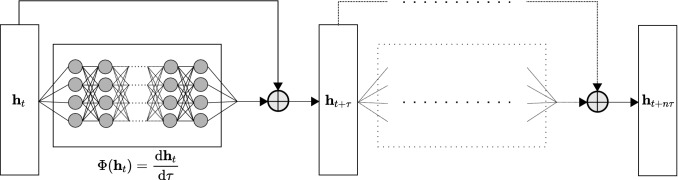
Neural ODE: For a dynamical system, we discretize a single step into *n* intervals of time $\tau$. For each interval, we use a Multi Layer Perceptron (MLP) to approximate the forward derivative. Using a residual connection, we can approximate a forward integration for a time interval $\tau$. We repeat this process *n* times for the full NODE.

The optimization for NODEs relies on the *adjoint sensitivity method* by Pontryagin et al.^44^. For a dataset generated by the underlying state-space function *u*(*t*) and a model $\Phi$ of parameters $\psi$ of size $\Psi$ replicating the flow of *u*(*t*), the initial value problem (IVP) can be formulated as:

11 $$\begin{aligned} \text {IVP:} {\left\{ \begin{array}{ll} \frac{\text {d}u(t)}{\text {d}t} = \Phi (u, t, \psi )\\ u(t=0) = u_0 \end{array}\right. } \end{aligned}$$

The model $\Phi$ is optimized by minimizing the loss $\mathfrak {J}$ over the time horizon *T*. Here $\mathfrak {J}$ is defined as:

12 $$\begin{aligned} \mathfrak {J}(u, \psi ) = \int _0^T g(u, \psi ) \text {d}t \end{aligned}$$

Where *g* is an arbitrary quadrature computing the fitness between $\frac{\text {d}u(t)}{\text {d}t}$ and $\Phi (u, t, \psi )$. In the case of NODEs, the model $\Phi$ is a neural network optimized through backpropagation. Backpropagation requires the computation of the gradients of the loss with respect to the parameters, i.e. :

13 $$\begin{aligned} \frac{\text {d}\mathfrak {J}}{\text {d}\psi }(u, \psi ) = \int _0^T \frac{\text {d}}{\text {d}\psi } g(u, \psi ) \text {d}t = \int _0^T \frac{\partial g}{\partial \psi }(u, \psi ) + \frac{\partial g}{\partial u}(u, \psi ) \frac{\text {d} u}{\text {d}\psi }(t) \text {d}t \end{aligned}$$

The $\frac{\text {d} u}{\text {d}\psi }(t)$ term scales linearly with the number of parameters $\Psi$. For a single parameter $\psi _i, i \le \Psi$, the following IVP must be satisfied:

14 $$\begin{aligned} \text {IVP:} {\left\{ \begin{array}{ll} \frac{\text {d}}{\text {d}\psi _i} \frac{\text {d}u(t)}{\text {d}t} = \frac{\text {d}}{\text {d}\psi _i} \Phi (u, t, \psi )\\ \frac{\text {d}}{\text {d}\psi _i} u(t=0) = \frac{\text {d}}{\text {d}\psi _i} u_0 \end{array}\right. } \end{aligned}$$

Thus every single additional parameter will result in an additional IVP that will need to be solved jointly with the initial IVP. This makes the implicit solving (Euler, Runge-Kutta, etc...) of the IVP unfeasible with models such as neural networks where $\Psi \gg 1$.

Pontryagin et al.’s^44^ *adjoint sensitivity method* proposes to solve the IVP using the Lagrange multiplier method over time with the model fitness as a constraint. The optimization Lagrangian $\mathcal {L}$ with multipliers $\lambda$ is thus given as:

15 $$\begin{aligned} \mathcal {L}(u, \lambda , \psi ) = \mathfrak {J}(u, \psi ) + \int _0^T \lambda ^T(t) (\Phi (u, t, \psi ) - \frac{\text {d}u(t)}{\text {d}t}) \text {d}t \end{aligned}$$

The multipliers $\lambda$ are constrained such that the $\frac{\text {d} u}{\text {d}\psi }(t)$ are negated from the gradient of the Lagrangian. The computation of $\lambda$ for all *t* is itself the result of a terminal value problem (TVP) given as:

16 $$\begin{aligned} \text {TVP:} {\left\{ \begin{array}{ll} \frac{\text {d}\lambda ^T(t)}{\text {d}t} = -\frac{\partial \Phi }{\partial u}(u, t, \psi ) \lambda ^T(t)\\ \lambda ^T(t=T) = - \frac{\partial \mathfrak {J}}{\partial u}(u, \psi ) \end{array}\right. } \end{aligned}$$

Therefore during the computation of the Lagrangian, we only need to call our implicit solvers twice: once for the original IVP, and once for the multipliers’ TVP solving backwards in time. The remaining gradient terms $\frac{\partial \Phi }{\partial u}$, $\frac{\partial \Phi }{\partial \psi }$, $\frac{\partial g}{\partial u}$, $\frac{\partial g}{\partial \psi }$ and $\frac{\partial u_0}{\partial \psi }$ can be computed numerically using reverse automatic differentiation. The NODE’s optimization can be summarized by the following steps:

- Solve the IVP for the initial *u*(*t*).
- Compute $$\lambda ^T(t=T)$$.
- Solve the TVP for $\lambda (t)$.
- Compute gradients and perform backpropagration.

The overall computation flow of the NODE is summarized in Fig. 4. Chen et al.^15^ and Rubanova et al.^23^ have shown that such a paradigm can further be applied with a generative approach; from a set of samples $\textbf{x}$, a latent variable space $\textbf{z}$ can be learned. This variable can then be evolved into future time steps to generate future predictions. This is equivalent to the standard VAE setting where the latent space can be evolved forward in time to generate future data samples. Using a decoder with parameters $\theta$ and using the encoder parameters $\phi$ to replace $\psi$, the NODE in the VAE context is given by:

17 $$\begin{aligned} \textbf{z}_{\tau _0} \sim p(\textbf{z}_{\tau _0}) \end{aligned}$$

18 $$\begin{aligned} \textbf{z}_{\tau _1}, \textbf{z}_{\tau _2},..., \textbf{z}_{\tau _N} = \text {ODESolve}(\textbf{z}_{\tau _0}, \Phi , \phi , \tau _0,..., \tau _n) \end{aligned}$$

19 $$\begin{aligned} \text {each} \ \ \textbf{x}_{\tau _i} \sim p(\textbf{x} \vert \textbf{z}_{\tau _i}, \theta ) \end{aligned}$$

In their approach, the NODE was used to obtain a decoder capable of generating observations for an irregularly sampled time-series. In contrast we make use of NODEs as en encoder for a better approximation of the a posteriori distribution.

### Hamiltonian neural networks

An emerging field in Machine Learning is that of Physics-Informed Neural Networks^24^. The intuition behind such networks is to add additional biases to Machine Learning models, such as conservation and invariance, to incorporate the physical laws that govern the observed system in the first place. Greynanus et al.^34^ made the observation that such laws can be described within the framework of Hamiltonian mechanics. At any arbitrary time step, the update of the position $\textbf{q}$ and the momentum $\textbf{p}$ for a Hamiltonian system described by the Hamiltonian function $\mathcal {H}$ can be given as:

20 $$\begin{aligned} \frac{d\textbf{q}}{dt} = \frac{\partial \mathcal {H}}{\partial \textbf{p}} \end{aligned}$$

21 $$\begin{aligned} \frac{d\textbf{p}}{dt} = -\frac{\partial \mathcal {H}}{\partial \textbf{q}} \end{aligned}$$

Based on the Hamiltonian, we can derive a symplectic forward map for the position and momentum. A mapping in a 3D space is symplectic^45^ if it induces volume preservation. The idea behind the method by Greydanus et al.^34^ is to parametrize the Hamiltonian by a neural network so as to embed the properties of symplecticity on the updates of the system. A similar method is that of Saemundsson et al.^35^. In this approach, the idea of symplecticity is introduced through the Lagrangian perspective. Assuming the mass to be retrievable, we only need to parametrize the potential energy for the forward step, known as the velocity-verlet integrator, as explained later. We justify our assumptions from the fact that in practical applications, one can usually estimate the mass with much more accuracy than the spring constants of the dynamical system being studied. For an autonomous non-dissipative system, the Euler-Lagrange equations are given as follows:

22 $$\begin{aligned} \mathfrak {L}(\textbf{q}, \dot{\textbf{q}}) = \mathfrak {T}(\dot{\textbf{q}}) - \mathfrak {U} (\textbf{q}) = \frac{1}{2} \dot{\textbf{q}}^{\textrm{T}} \textbf{M} \dot{\textbf{q}} - \mathfrak {U}(\textbf{q}) \end{aligned}$$

with $\textbf{q}$ denoting the vector of generalized coordinates, $\textbf{M}$ the diagonal mass matrix and $\mathfrak {T}$, $\mathfrak {U}$ denoting the kinetic and potential energy, respectively. The discrete time Lagrangian can be approximated, similarly to the Euler method, by integrating the equations of motion over an arbitrarily small time step *h*, i.e.:

23 $$\begin{aligned} \mathfrak {L}^d (\textbf{q}_t, \textbf{q}_{t+1}, h) \approx \int _{t}^{t+h} \mathfrak {L} \left(\textbf{q}(\tau ), \dot{\textbf{q}}(\tau )\right) \ d\tau \end{aligned}$$

Note that the above equation can be derived on the basis of the principle of least action (any real path is a sum of infinitesimal Lagrangian steps).

From this quadrature, we derive what are known as Variational Integrators (VI). VIs are an alternative to Euler-based methods for the numerical integration of Hamiltonian systems. VIs are known as symplectic integrators, since they conserve the continuous time energy of the system in the discrete domain with a third order error. VIs have been shown to be more stable, even for larger time steps which are not feasible for implicit integration methods^45^.

We derive our integrator as follows. We start by parametrizing the continuous Lagrangian with the parameter vector $\mathbf {\phi }$.

24 $$\begin{aligned} \mathfrak {L}_{\mathbf {\phi }}(\textbf{q}, \dot{\textbf{q}}) = \mathfrak {T}_{\mathbf {\phi }}(\dot{\textbf{q}}) - \mathfrak {U}_{\mathbf {\phi }}(\textbf{q}) = \frac{1}{2} \dot{\textbf{q}}^{\textrm{T}} \textbf{M}_{\mathbf {\phi }} \dot{\textbf{q}} - \mathfrak {U}_{\mathbf {\phi }}(\textbf{q}) \end{aligned}$$

A quadrature rule is applied to derive the discretized equivalent; here the trapezoidal rule is adopted:

25 $$\begin{aligned} \mathfrak {L}^d_{\phi }(\textbf{q}_t, \textbf{q}_{t+1}, h) = \frac{h}{2} \left(\mathfrak {L}_{\phi }\left(\textbf{q}_t, \frac{(\textbf{q}_{t+1} - \textbf{q}_t)}{h}\right) + \mathfrak {L}_{\phi } \left(\textbf{q}_{t+1}, \frac{(\textbf{q}_{t+1}-\textbf{q}_t)}{h}\right)\right) \end{aligned}$$

We can then derive the velocity-Verlet integrator, as per Saemundsson et al.^35^ :

26 $$\begin{aligned} \textbf{q}_{t+1} = \textbf{q}_t + h \textbf{M}_{\phi }^{-1} \dot{\textbf{q}}_t - \frac{h^2}{2} \textbf{M}_{\phi }^{-1} \frac{\partial \mathfrak {U}_{\phi }(\textbf{q}_t)}{\partial \textbf{q}_t} \end{aligned}$$

27 $$\begin{aligned} \textbf{p}_{t+1} = \textbf{p}_t - \frac{h}{2}\left(\frac{\partial \mathfrak {U}_{\phi }(\textbf{q}_t)}{\partial \textbf{q}_t} + \frac{\partial \mathfrak {U}_{\phi }(\textbf{q}_{t+1})}{\partial \textbf{q}_{t+1}}\right) \end{aligned}$$

with $$\textbf{p}_t = \textbf{M}_{\phi }^{-1} \dot{\textbf{q}_t}$$.

Saemundsson et al.^35^ then combine these symplectic steps into a single RNN to create a symplectic version of the NODE described above. This method was shown capable of retrieving the latent space for MDOF dynamical systems.

### Symplectic DVAE

**Figure 5:**
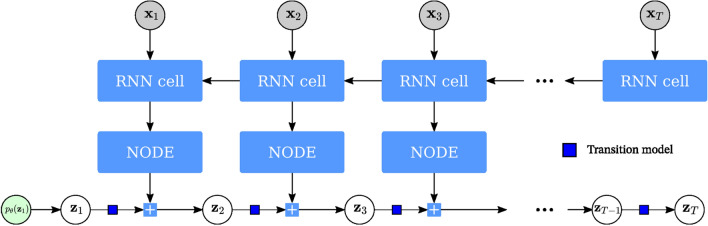
RNN-NODE encoder: Observations at time *t* are augmented to the latent space dimension using a RNN network. We use an RNN instead of a regular neural network so as to pass long-term dependencies to the next time step. The lifted data is then passed through a NODE to simulate a forward integration to estimate the next latent step. The NODE is modified such that its estimated flow is symplectic.

We combine all the models mentioned above into a symplectic DVAE. We do this by replacing the DMM’s RNN encoder with a NODE as per Fig. 5. It is noted here that we still use an RNN to augment the observations to the latent space’s dimension. Then, we pass these samples through the Neural ODE to predict the previous latent distribution; we pass the samples in reverse, since we wish to infer the posterior distribution. This process is summarized in Fig. 5. Furthermore, each layer of the NODE follows the Velocity-Verlet integration step. The full model architecture comprises the following neural networks :

- The emission neural network, an MLP with $L_E$ layers of $N_E$ width estimating the emission PDF $p(\textbf{x}_t \vert \textbf{z}_t)$
- The transmission neural network, a two layer MLP with a width of $N_T$ estimating the transmission PDF $p(\textbf{z}_t \vert \textbf{z}_{t-1})$
- The RNN with $L_{\text {RNN}}$ layers for the computation of the approximate posterior $q(\textbf{z}_t \vert \textbf{x}_{t:T})$
- The energy neural network; an MLP with $L_P$ layers of $N_P$ width estimating $\frac{\partial \mathfrak {U}_{\phi }(\textbf{q}_{t})}{\partial \textbf{q}_t}$

Our methodology can be summarized as follows: we postulate that our data is a random variable generated by a latent physical process which motivates our use of Variational Inference to learn a generative model. We choose the DMM as our generative model to enforce a Markov property which holds true globally for linear systems and locally for nonlinear systems. We use a NODE as our inference network to which biases the model to learn the flow of a dynamical system governed by an ODE. We can enforce additional conditions on the type of ODE that is learned. In our work, we choose to enforce symplectic constraints on the forward integration step to imbue our model with energy-preserving properties.

### Dissipative systems

The use of symplectic integration restricts us to the study of systems with constant total energy. However, the vast majority of systems with applications in industry are either non-autonomous, or dissipative, or both. In the context of this paper, we will focus on the latter. To incorporate the idea of dissipation into our system, we implemented a presymplectic Verlet integrator following the recommendations of Franca et al.^46^. By augmenting the integrator state with an auxiliary time variable $\tau _t$, we obtain the following updates for a single step:

28 $$\begin{aligned} \textbf{q}_{t+1} = \textbf{q}_t + h \textbf{M}_{\phi }^{-1} \dot{\textbf{q}}_t - \frac{h^2}{2} \textbf{M}_{\phi }^{-1} \frac{\partial \mathfrak {U}_{\phi }(\tau _t, \textbf{q}_t)}{\partial \textbf{q}_t} \end{aligned}$$

29 $$\begin{aligned} \tau _{t+1} = \tau _t + h \end{aligned}$$

30 $$\begin{aligned} \textbf{p}_{t+1} = \textbf{p}_t - \frac{h}{2}\left(\frac{\partial \mathfrak {U}_{\phi }(\tau _t, \textbf{q}_t)}{\partial \textbf{q}_t} + \frac{\partial \mathfrak {U}_{\phi }(\tau _{t+1}, \textbf{q}_{t+1})}{\partial \textbf{q}_{t+1}}\right) \end{aligned}$$

where, as previously, with $\textbf{p}_t = \textbf{M}_{\phi }^{-1} \dot{\textbf{q}_t}$.

## Experiments

We implement both the emitter and transmission MLP using the popular PyTorch library. The encoder’s RNN is also implemented using PyTorch^47^. For the neural ODE, we use a modified version of the torchdiffeq library^15^ provided by Ishikawa^48^. For the variational inference, we rely heavily on the automated processes provided by the Pyro library^49^. Notably, we let the library handle the calculation of the ELBO as well as all Monte Carlo simulations necessary for stochastic backpropagation.

### Dynamical system

The focus on our experiments will be the study of linear and nonlinear ODE systems subject to noise. The ODE governing the dynamics of each system is defined as:

31 $$\begin{aligned} \textbf{M} \ddot{\textbf{q}}(t) + \textbf{C} \dot{\textbf{q}}(t) + K(\textbf{q}(t)) = D(t) \end{aligned}$$

Where $\textbf{M}$ is the diagonal mass matrix, $\textbf{C}$ is the dissipation matrix, *K* is the potential function, *D* is the external excitation force function and $\textbf{q}(t)$ is the position vector. In our default experiments, we assume that displacements and accelerations are measurable. For all systems, we assume that these measurements are available for all DOFs (in a separate section, we explore the case where part of the DOFs are not accessible.). We will also assume that the mass matrix is known ($\textbf{M} = \textbf{I}$ the identity matrix) and that the rest of the parameters for $\textbf{C}$, *K* and $\textbf{D}$ are randomly sampled from the uniform distribution $\mathcal {U}[0, 2]$. Furthermore, we make the assumption that displacement measurements are subject to higher uncertainty than that of accelerations, and therefore corrupt the former with 10dB of Gaussian noise and the latter with 20dB of Gaussian noise.

In each experiment, the training dataset is generated as follows. Once we have defined the parameters of the dynamical system, we solve the ODE using RK45^50^ for 500 iterations with different initial values. We then select our observed states (positions and accelerations) and corrupt these with additive Gaussian noise. We would like to note that we are aware that this assumption already poses some form of bias on the assumed noise distribution. However, non-Gaussian noise could be easily tackled via use of normalizing flows^51^. Inspired by Saedmunsson et al.^35^, we augment our dataset by subdividing each simulation into windows of 50 with a shift of 1.

### Coupled spring-mass system

**Figure 6:**
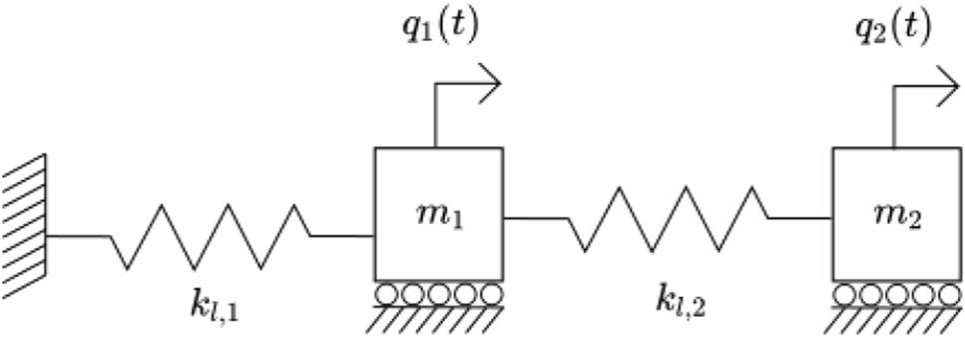
Autonomous 2DOF system.

As a proof of concept, we start off by studying the dynamics two masses coupled with two springs, as shown on Fig. 6. For now, we only consider the free vibration non-dissipative responses of the system. As observables, we choose the position and the acceleration of both masses, as these are the most common quantities measured my sensors in monitoring systems. We define the equations of motion for each degree of freedom and put these in matrix form, i.e. :

32 $$\begin{aligned} \textbf{M} \ddot{\textbf{q}}(t) + \textbf{K}_l \textbf{q}(t) = \textbf{0} \end{aligned}$$

Here, the potential function $K(\textbf{q}(t)) = \textbf{K}_l \textbf{q}(t)$, i.e. is a linear transformation. The excitation function $\textbf{D}(t) = \textbf{0}$ the zero matrix.

#### Optimal parameter search

Our model’s ability to fit the training data is highly dependant on the hyperparameters defining the dimensions of the different neural networks. However, these networks are relatively small ($N_{\text {parameters}} \sim 10^3$), which renders the involved training costs computationally cheap. Motivated by the low dimensionality of the problem, we perform a hyperparameter search using Bayesian Parameter Search^52^ to determine the optimal model configuration, which is found to be described by the following parameters:

**Table:** 

Parameter	$N_{E}$	$L_{E}$	$N_{T}$	$N_{P}$	$L_{P}$	$L_{\text {RNN}}$
Value	18	2	15	85	3	15

**Figure 7:**
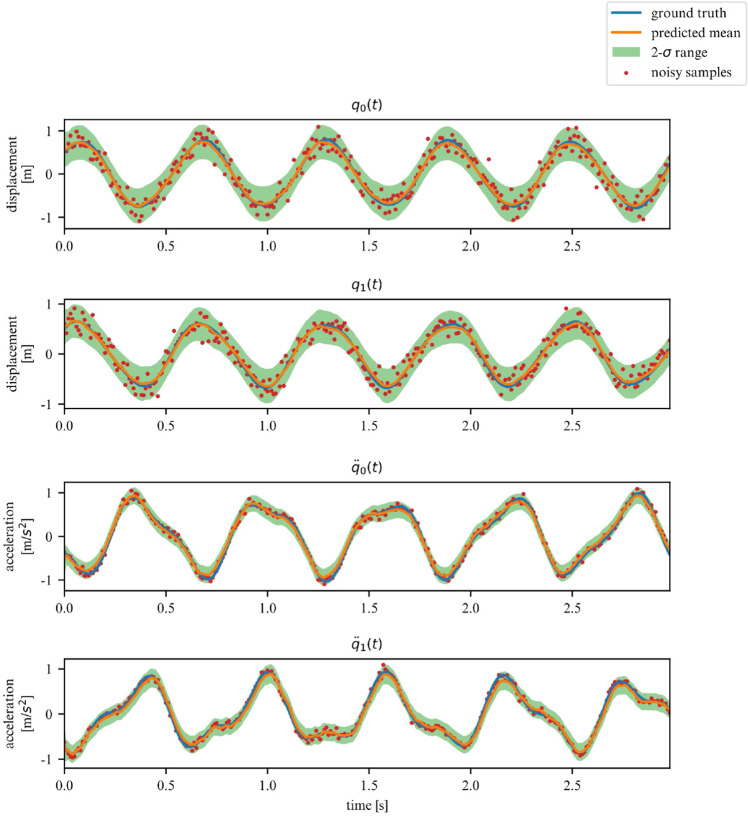
Observations for 2DOF system.

**Figure 8:**
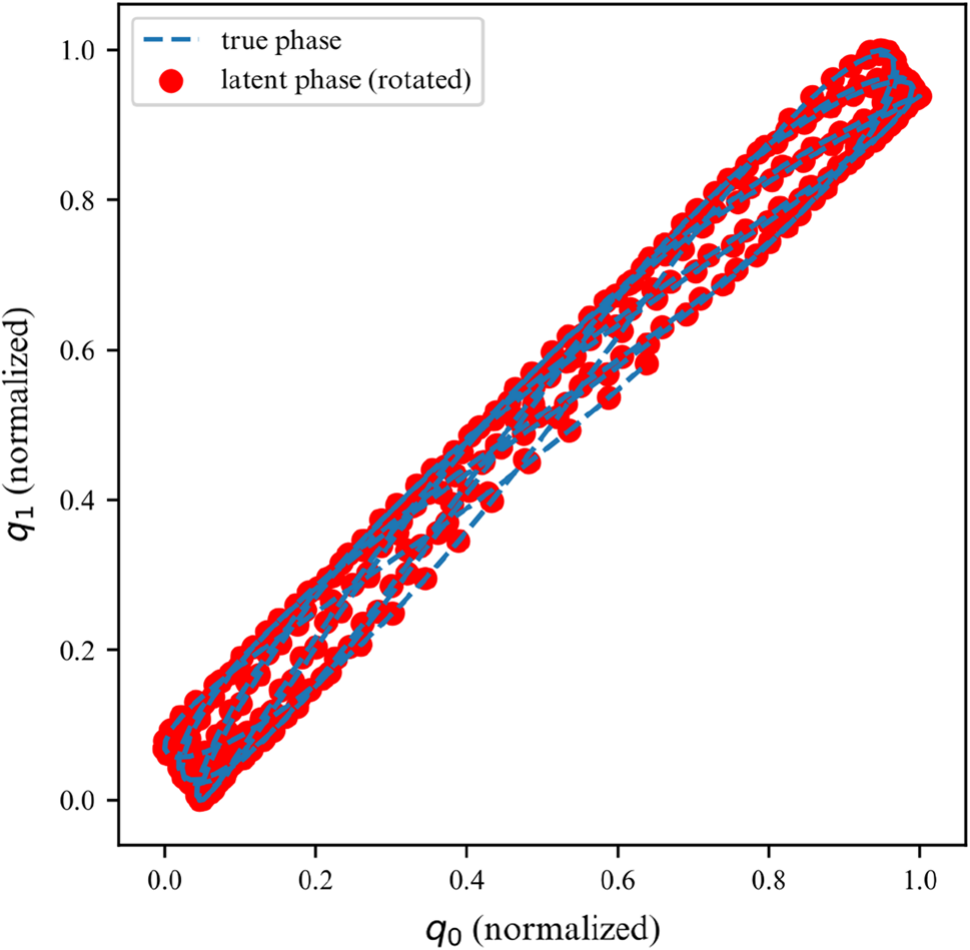
Phase space for first DOF of a linear 2DOF system.

While the ELBO allows our DMM to infer latent variables from noisy data, is does not offer an interpretable metric on how well the model fits the data. Once trained, we can use the derived DMM as a one-step ahead predictor to verify how well it has learned the dynamics of the system (Fig. 7). To further verify that the model has learned the underlying dynamics, we can plot the phase-space of the latent representation. In our case, the model learns a rotated version of the phase-space; symplecticity only constrains the area, not the orientation. Therefore, we can correct for the angle by applying a rotation transformation calculated with a least-square estimator. On Fig. 8 we can see that both phase-spaces line up, indicating that our model was able to learn a latent representation corresponding to the phase-space of the linear system.

### Linear system of higher dimensionality

Moving on, we investigate the ability of the proposed model to adapt to a higher number of degrees of freedom. We verify that our model can scale up by learning the dynamics of a 3DOF system. The optimal parameter search now returns the following hyperparameters:

**Table:** 

Parameter	$N_{E}$	$L_{E}$	$N_{T}$	$N_{P}$	$L_{P}$	$L_{\text {RNN}}$
Value	29	1	24	11	5	1

**Figure 9:**
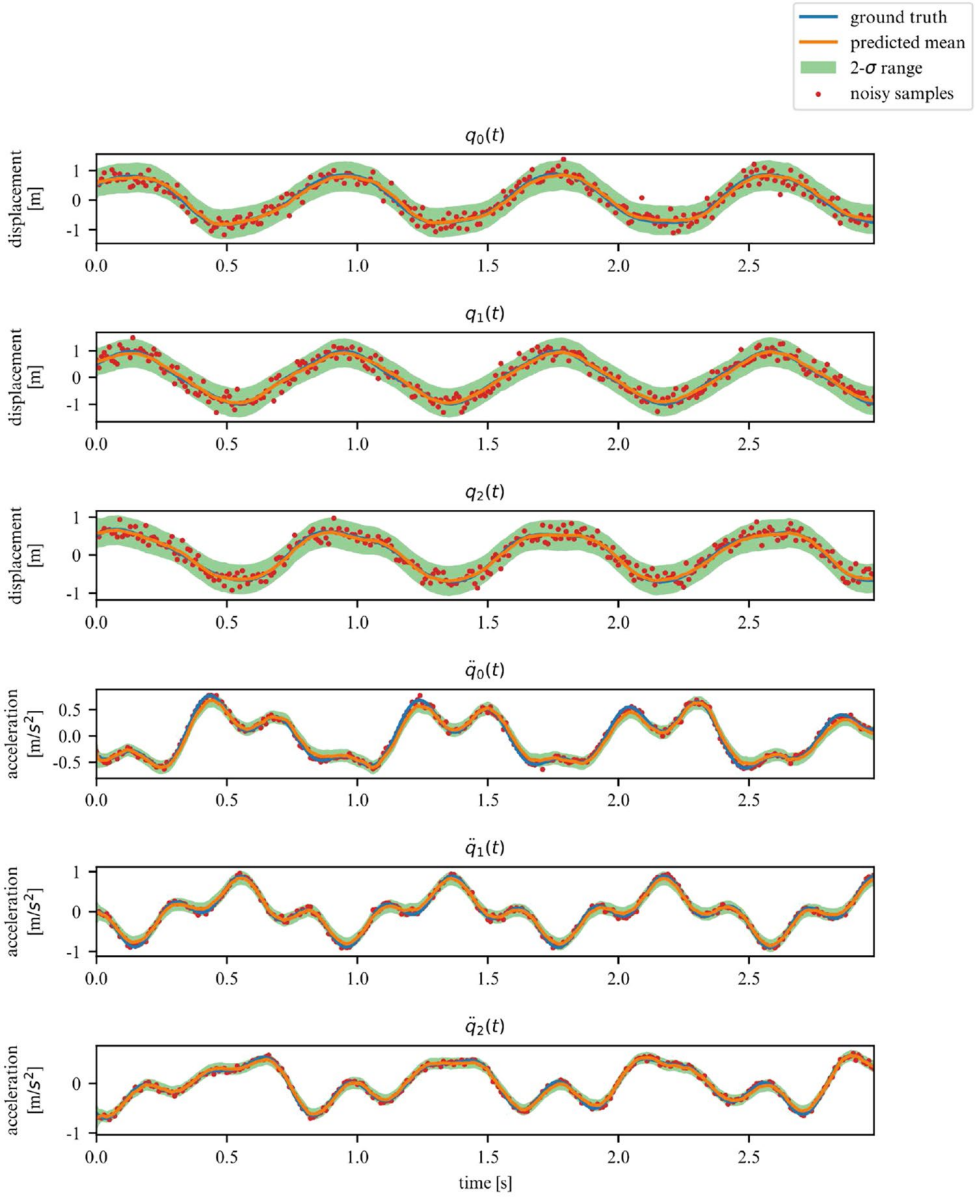
Observations for a linear 3DOF system.

**Figure 10:**
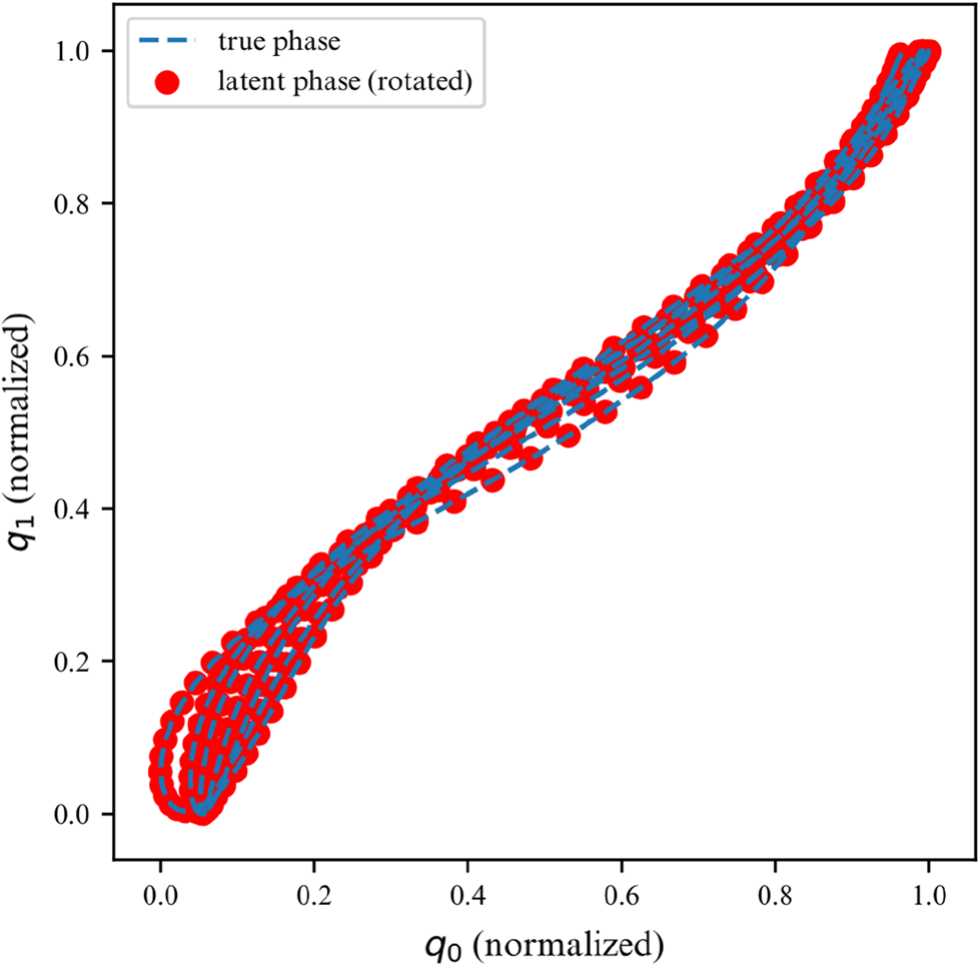
Phase space for the first DOF of a 3DOF system.

We corroborate our previous conclusions for the 3DOF system on Figs. 9 and 10.

While we have just demonstrated that the number of DOFs can be increased, our model struggles to scale for much larger numbers of DOFs. This is due to the heavy influence of our hyperparameters on the performance of the model. This makes the Bayesian Parameter Search more and more costly as the number of DOFs increases. We leave this weakness of our model as a topic for further research.

### Nonlinear dynamics

#### Two degree of freedom duffing oscillator

**Figure 11:**
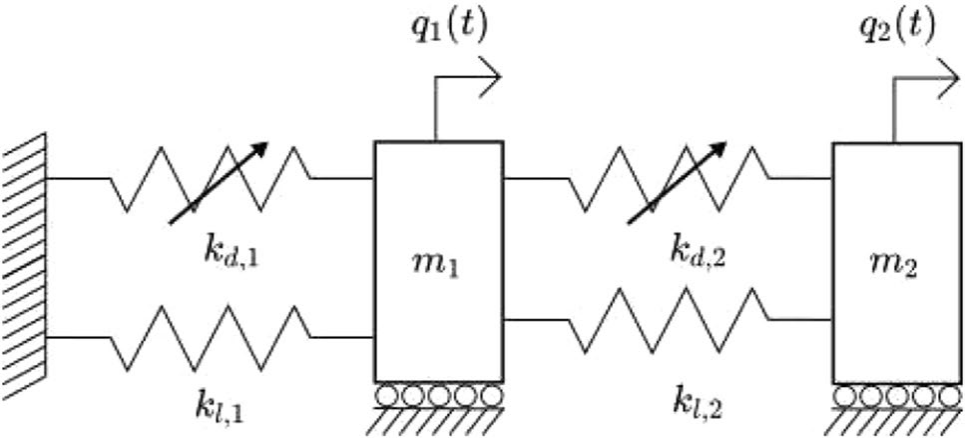
2DOF duffing oscillator system.

We modify the previous 2DOF example via addition of cubic springs, resulting in a two degree of freedom Duffing oscillator, as shown on Fig. 11.

This adds a cubic nonlinear term to the elastic potential. The updated equations of motion are now given by :

33 $$\begin{aligned} \textbf{M} \ddot{\textbf{q}}(t) + \textbf{K}_l \textbf{q}(t) + \textbf{K}_d \textbf{q}^3(t)= \textbf{0} \end{aligned}$$

Here, the potential function $K\left(\textbf{q}(t)\right) = \textbf{K}_l \textbf{q}(t) + \textbf{K}_d \textbf{q}^3(t)$, i.e. is a nonlinear transformation. Nonlinear dynamical systems are notorious for exhibiting chaotic dynamics for certain regions of the initial phase-space. To explore the impact of the initial conditions on the dynamics of this system, we calculate the Finite-Time Lyapunov Exponent (FTLE) along the phase-plane. The FTLE is a useful metric to quantify the deviation of the trajectory of the system for an infinitesimal perturbation around a given initial condition. A high FTLE would indicate a high deviation for a small perturbation, which is a characteristic of a chaotic trajectory. The FTLE is given by:

34 $$\begin{aligned} \text {FTLE}_{t_0}^{T}(\textbf{q}_0) = \frac{1}{2 \vert T-t_0 \vert } \log \left( \omega _{\text {max}} (\nabla _{\textbf{q}} \Phi ^{\textrm{T}}(\textbf{q}_0) \nabla _{\textbf{q}} \Phi (\textbf{q}_0))\right) \end{aligned}$$

with $\textbf{q}(T) = \Phi (\textbf{q}_0)$ and $\omega _{\text {max}}$ a function that returns the maximum eigenvalue of the input. We can now calculate the FTLE for both DOFs along the [-2, 2] square phase-space with an initial $\Delta _{q_0} = \Delta _{\dot{q}_0} = 0.01$:

**Figure 12:**
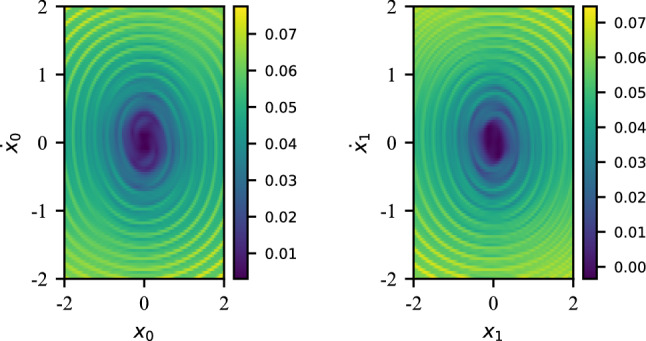
FTLE for the duffing oscillator.

An observation of Fig. 12 indicates that as we move away from the origin along the phase-space, the FTLE tends to increase, particularly around “chaotic rings”. Running a hyperparameter sweep, we are able to design a model capable of adapting to this more diverse dataset:

**Table:** 

Parameter	$N_{E}$	$L_{E}$	$N_{T}$	$N_{P}$	$L_{P}$	$L_{\text {RNN}}$
Value	14	1	36	54	1	2

**Figure 13:**
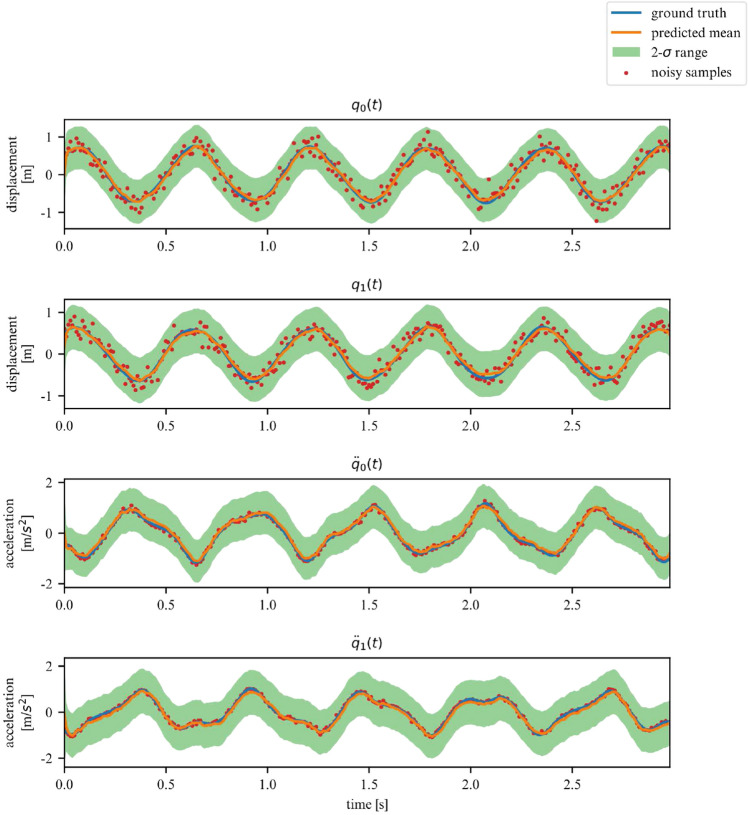
Duffing oscillator observations.

**Figure 14:**
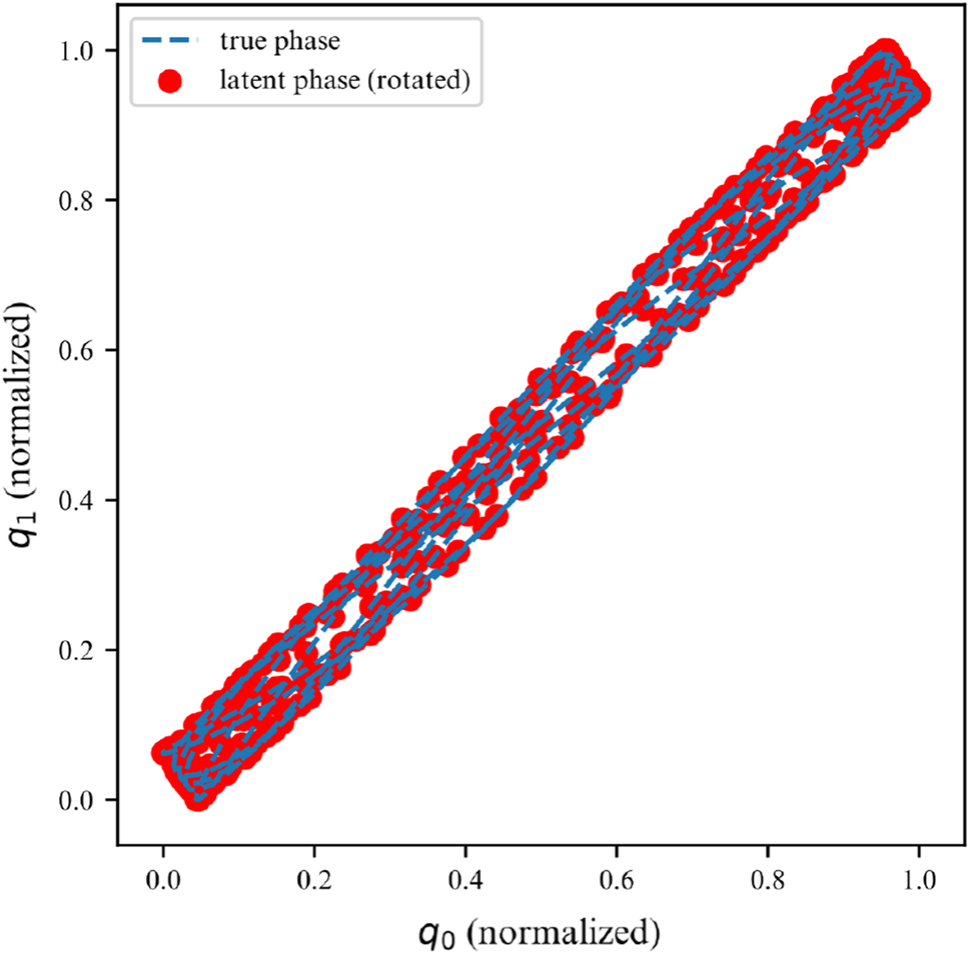
Duffing oscillator phase space.

Figures 13 and 14 verify the ability of the proposed model to learn nonlinear dynamics just like in the linear dynamics. In the case of the Duffing oscillator, the chaoticness is low enough for our model to generalize for the entire phase-space.

#### Double pendulum

**Figure 15:**
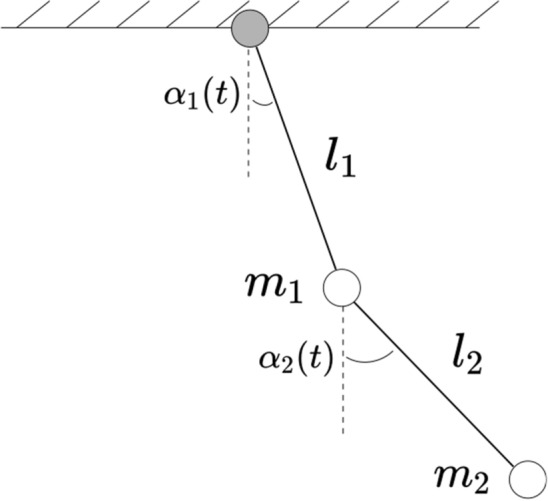
Double pendulum system.

The NDOF pendulum is another example of a nonlinear system that exhibits chaotic dynamics. Already for a 2DOF pendulum (Fig. 15), the equations of motion are quite complicated. Using angular coordinates $\mathbf {\alpha }$ for the angle and $\textbf{l}$ for the length, the dynamics is described as follows:

35 $$\begin{aligned} {\left\{ \begin{array}{ll} (m_1 + m_2) l_1 \ddot{\alpha _1} + m_2 l_2 \ddot{\alpha _2} \cos (\alpha _1 - \alpha _2) + m_2 l_2 \dot{\alpha _2}^2 \sin (\alpha _1-\alpha _2) + (m_1 + m_2) \mathcal {G} \sin (\alpha _1) = 0 \\ m_2 l_2 \ddot{\alpha _2} + m_2 l_1 \ddot{\alpha _1} \cos (\alpha _1 - \alpha _2) - m_2 l_1 \dot{\alpha _1}^2 \sin (\alpha _1 - \alpha _2) + m_2 \mathcal {G} \sin (\theta _2) = 0 \end{array}\right. } \end{aligned}$$

**Figure 16:**
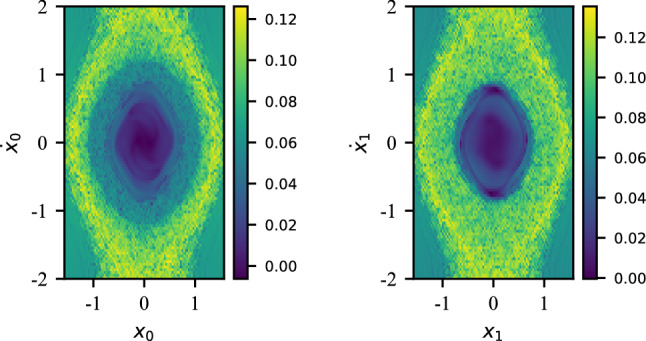
FTLE for the double pendulum.

In our study, we will fix $m_1 = m_2 = 1$ and $l_1 = l_2 = 8$ and $\mathcal {G}$ is the gravitational constant. We compute the FTLE for the $[-\pi /2, \pi /2, -2, 2]$ phase-space of the pendulum with an initial $\Delta _{x_0} = \Delta _{\dot{x}_0} = 0.01$ (Fig. 16).

Unlike the duffing oscillator, the double pendulum’s phase-space exhibit chaotic dynamics in broader regions, typically when $\theta _2 > \frac{\pi }{2}$. The proposed models is not able to generalize for such as diverse set of dynamics. Focusing on a subs-space of the phase-space, namely [0, 1, 0, 1], we were able to train a model via use of a hyperparameter sweep :

**Table:** 

Parameter	$N_{E}$	$L_{E}$	$N_{T}$	$N_{P}$	$L_{P}$	$L_{\text {RNN}}$
Value	13	0	18	21	4	1

**Figure 17:**
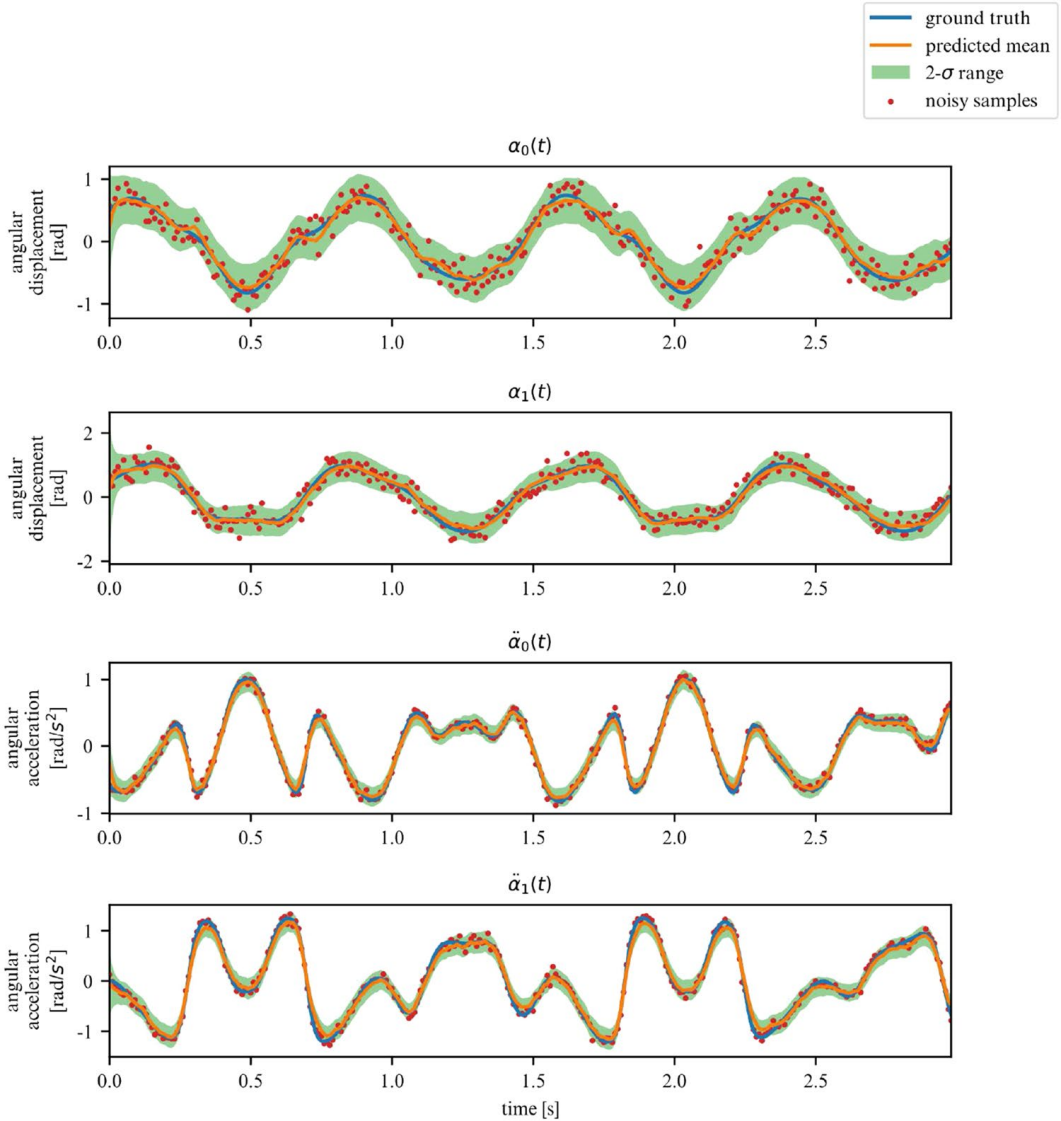
Double pendulum observations.

**Figure 18:**
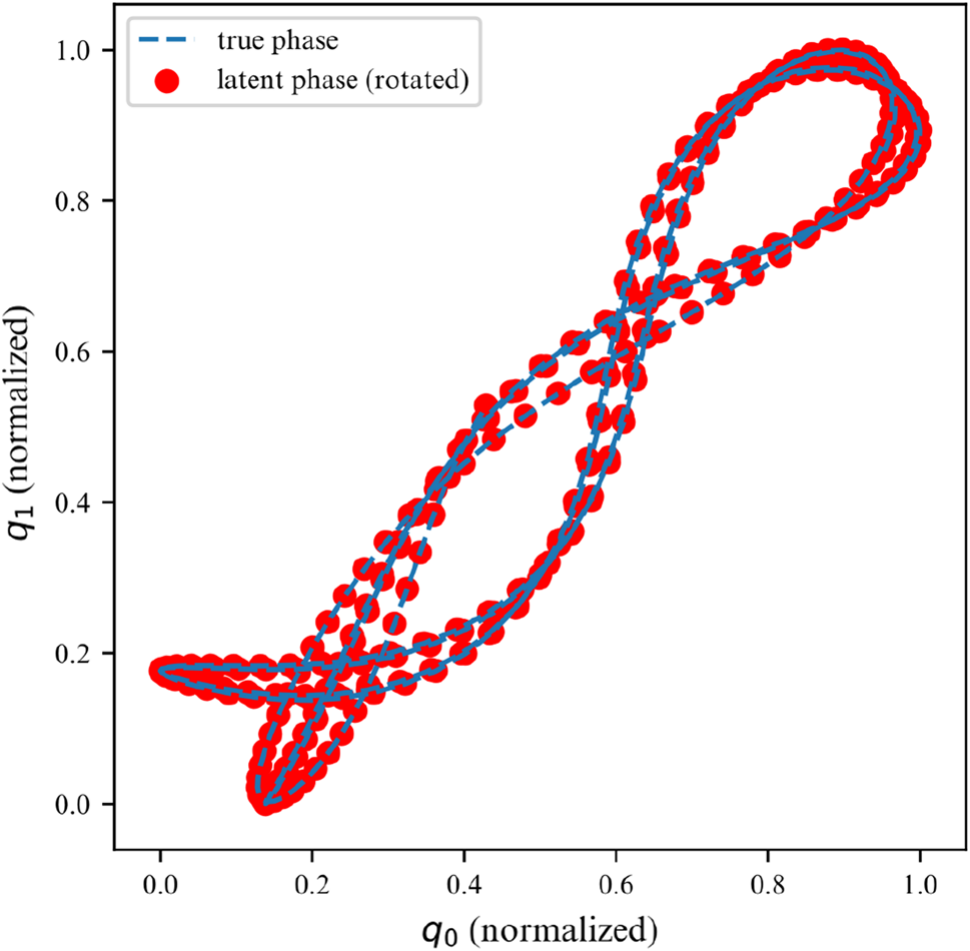
Double pendulum phase space.

Over a limited non-chaotic phase-space, the dynamics of the double pendulum can be learned, as per Figs. 17 and 18. This demonstrates versatility of the proposed model, in the sense that it can be repurposed for a variety of nonlinear dynamics, as long as the phase-space only contains few chaotic initial configurations.

### Systems with partial observations

**Figure 19:**
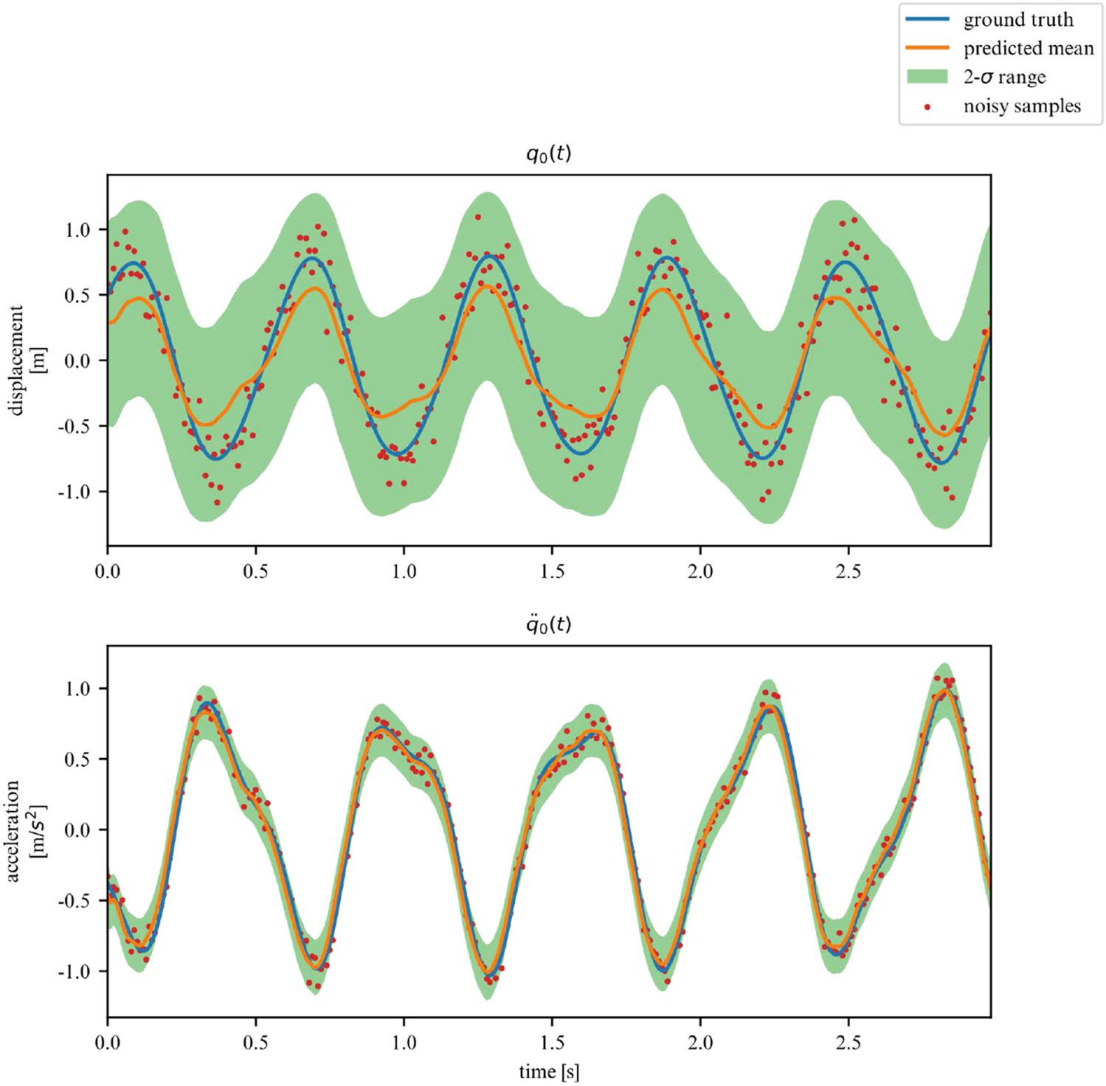
Partially observed linear 2DOF system.

**Figure 20:**
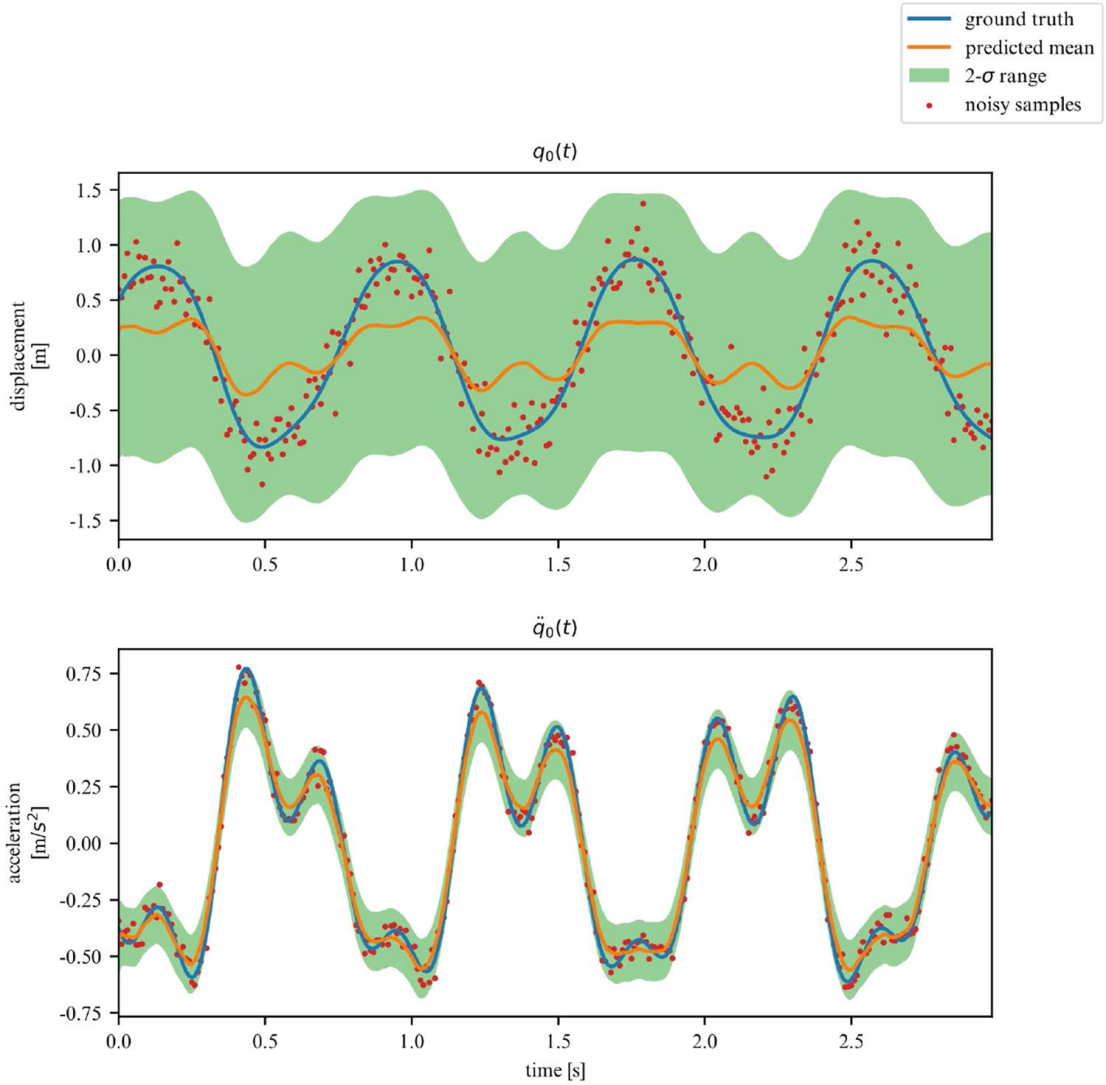
Partially observed linear 3DOF system.

**Figure 21:**
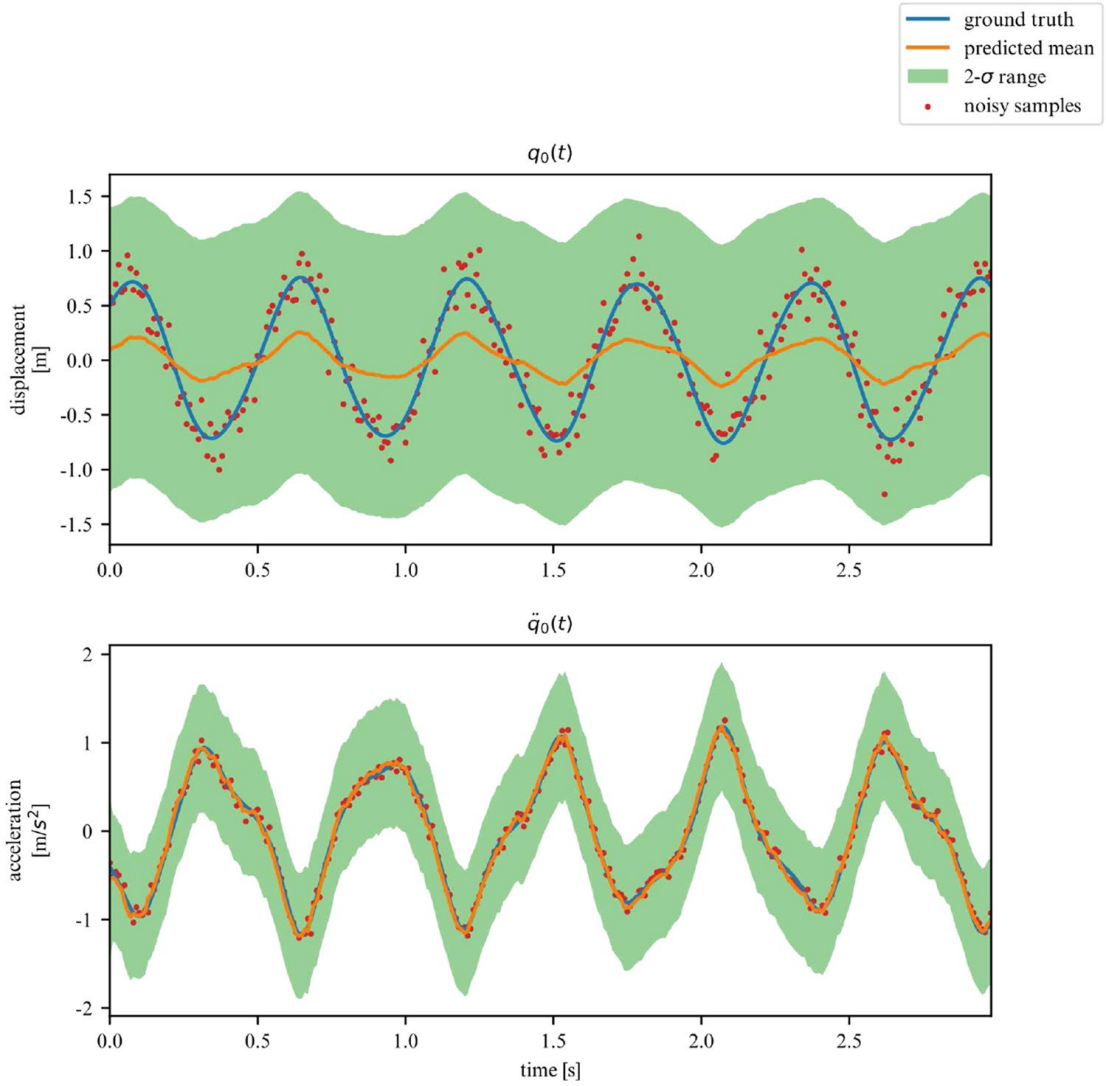
Partially observed duffing 2DOF system.

**Figure 22:**
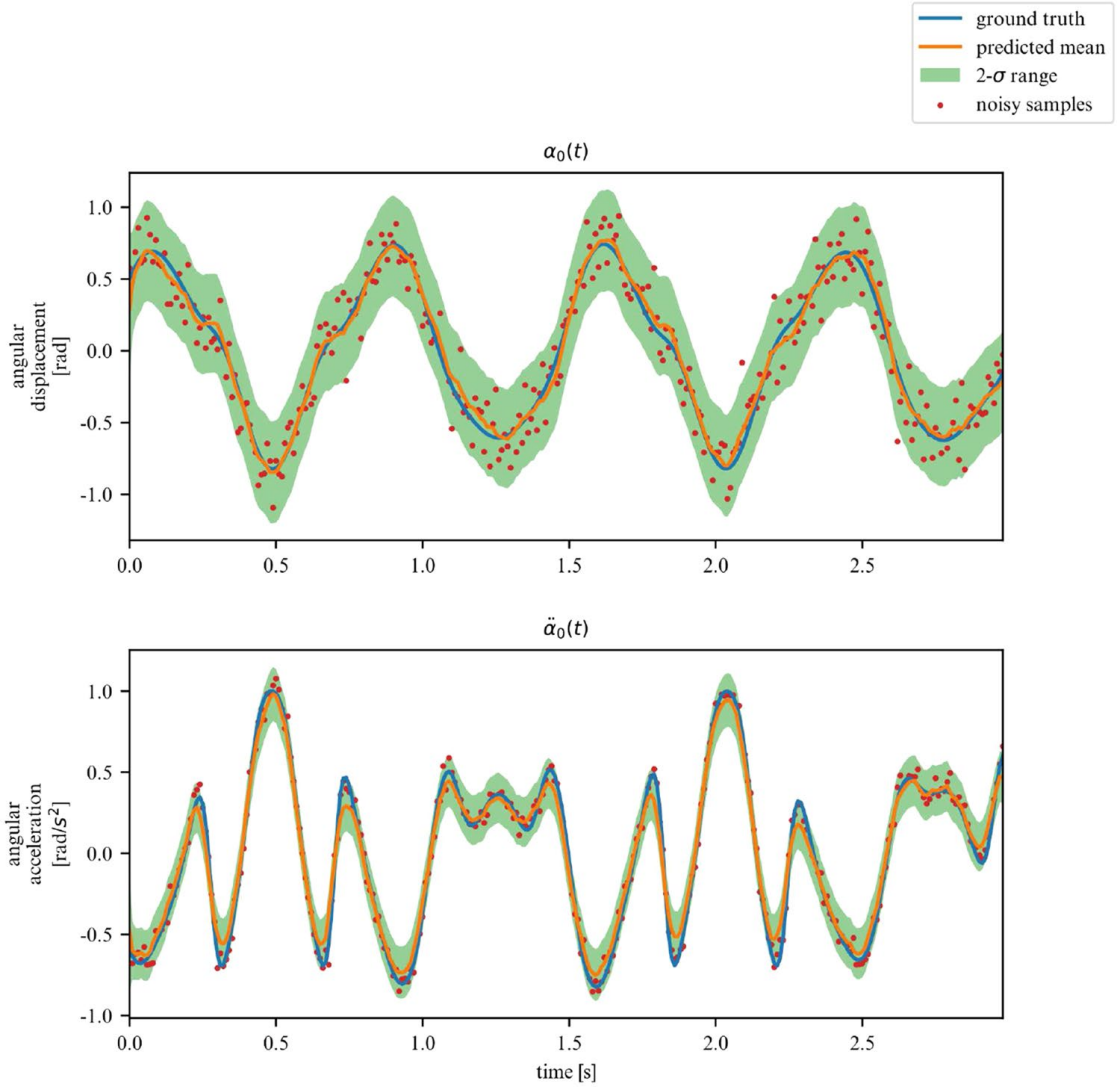
Partially observed double pendulum system.

While we deemed it useful for demonstration purposes in what was shown in the previous examples, the proposed framework does not require the observation of all DOFs. In this section, we demonstrate its performance on learning the dynamics of the previously examined systems, but this time for a subset of observations. More specifically, we now chose to only observe the acceleration and displacement of the first degree of freedom. We apply this approach to the 2DOF linear, 3DOF linear, 2DOF duffing and 2DOF pendulum systems. Samples of the observed time series are showcased in Figs. 19, 20, 21 and 22.

In these scenarios, the model has to deal with a greater level of uncertainty, and this is reflected in the widening of the variance band, particularly for displacements. However, overall, our model is able to generalize for linear and nonlinear systems, even when only a subset of the system’s DOFs are observed (measured).

### Nonautonomous systems

#### Dissipation

We return to the coupled spring-mass system. However, we now add damping to both degrees of freedom. The corresponding equations of motion are now given by:

36 $$\begin{aligned} \textbf{M} \ddot{\textbf{q}}(t) + \textbf{C} \dot{\textbf{q}}(t) + \textbf{K}_l \textbf{q}(t) = \textbf{0} \end{aligned}$$

We retrain the same model as in section 4.2, but now with implementation of the dissipative integrator described in section 3.5.

**Figure 23:**
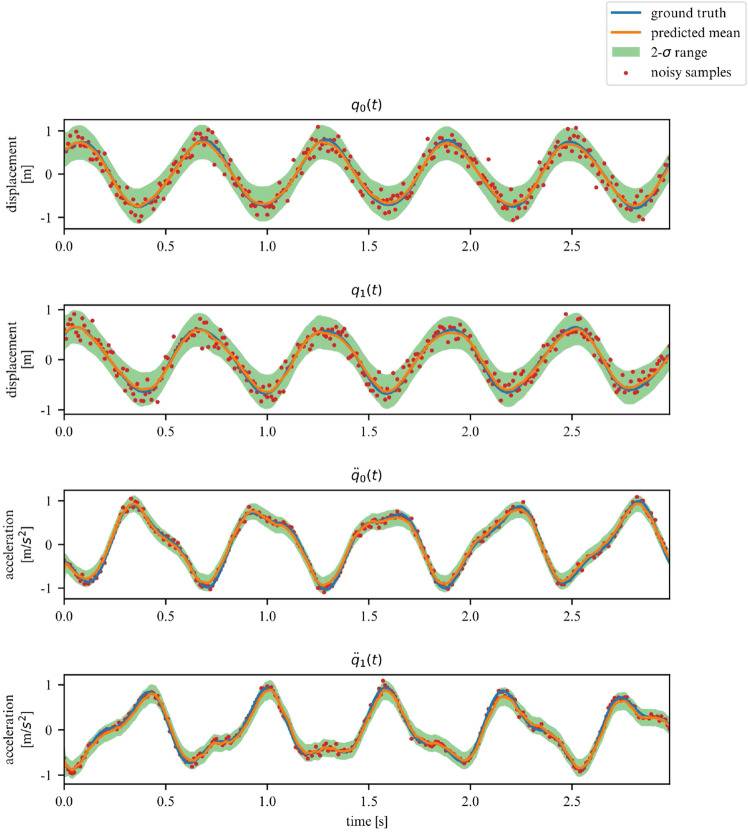
Linear dissipative 2DOF system observations.

**Figure 24:**
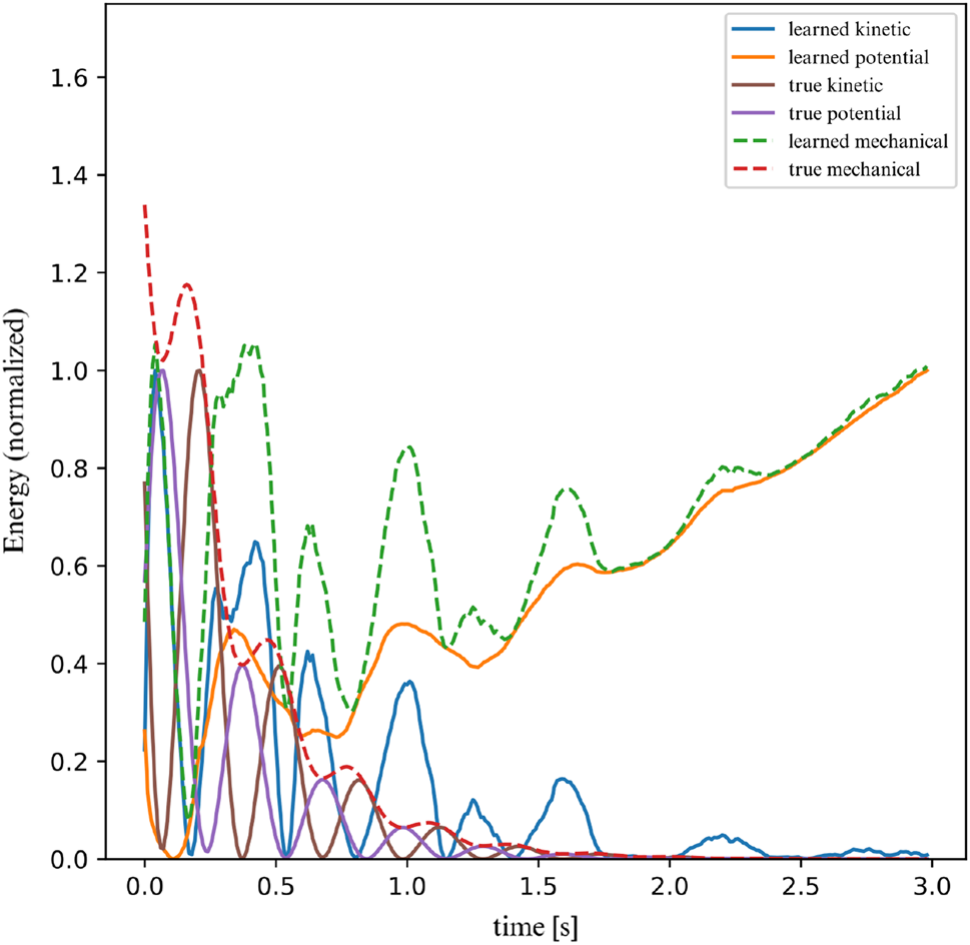
Linear dissipative 2DOF system energy.

Figure 23 illustrates that the herein proposed model delivers stable predictions on the dynamics of the model. However, we would further like to verify whether the model’s latent space takes into account the loss of energy of the system. The results obtained in Fig. 24 are mixed. The model reflects the fact that the energy variations are diminishing over time and the model is converging towards a constant state. However, we are left with a large residual energy even when the system is at rest. This is because of the gauge invariance of the potential energy of this system; a trajectory can give us information on the gradient of the energy, but there are infinitely many possible starting points. Since we do not offer any prior information on the initial potential energy to the model, it will not be able to recover this information by simply observing the system’s trajectories.

#### Forced dissipative nonlinear dynamics

We now additionally subject the dissipative version of a nonlinear system to external forces. In particular, we will study the effects of a sinusoidal excitation on a dissipative 2DOF duffing oscillator. The corresponding equations of motion are now given by:

37 $$\begin{aligned} \textbf{M} \ddot{\textbf{q}}(t) + \textbf{C} \dot{\textbf{q}}(t) + \textbf{K} \textbf{q}(t) + \textbf{K}_d \textbf{q}^3(t) = \textbf{D}\varvec{\gamma } \sin (2 \pi f t) \end{aligned}$$

**Figure 25:**
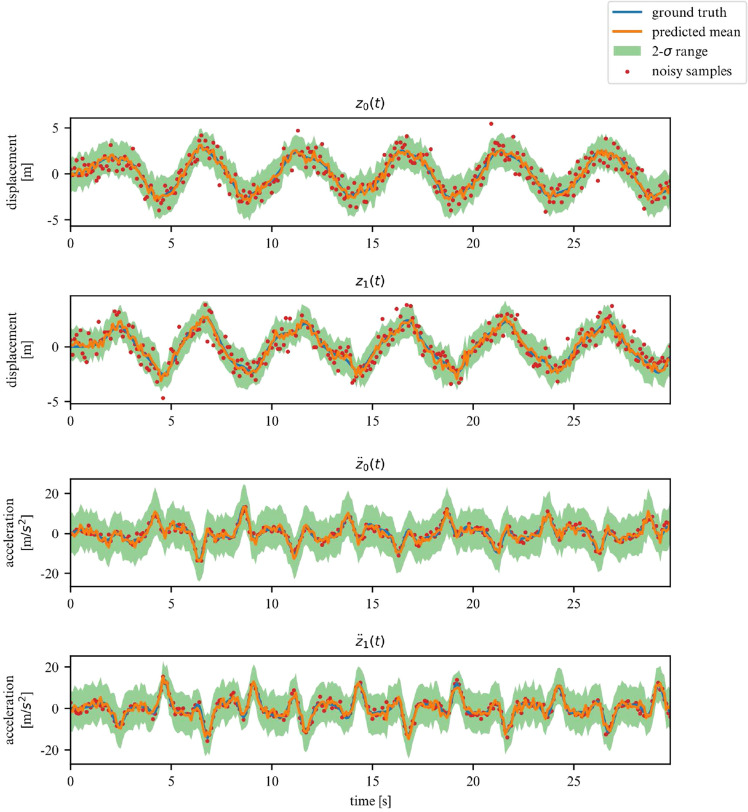
Forced dissipative 2DOF system observations for $\gamma = 0.3$.

**Figure 26:**
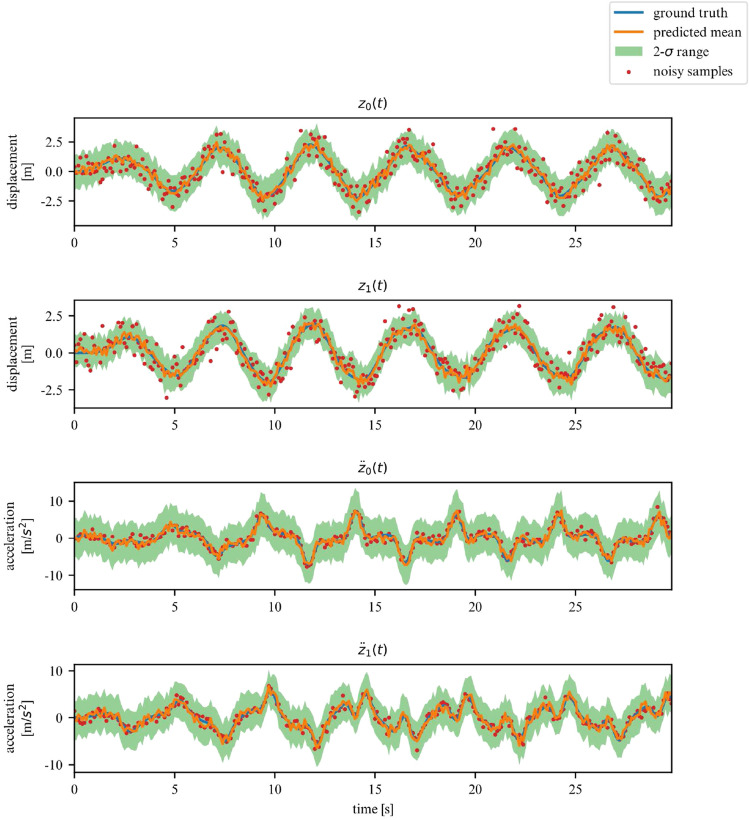
Forced dissipative 2DOF system observations for $\gamma = 0.9$.

**Figure 27:**
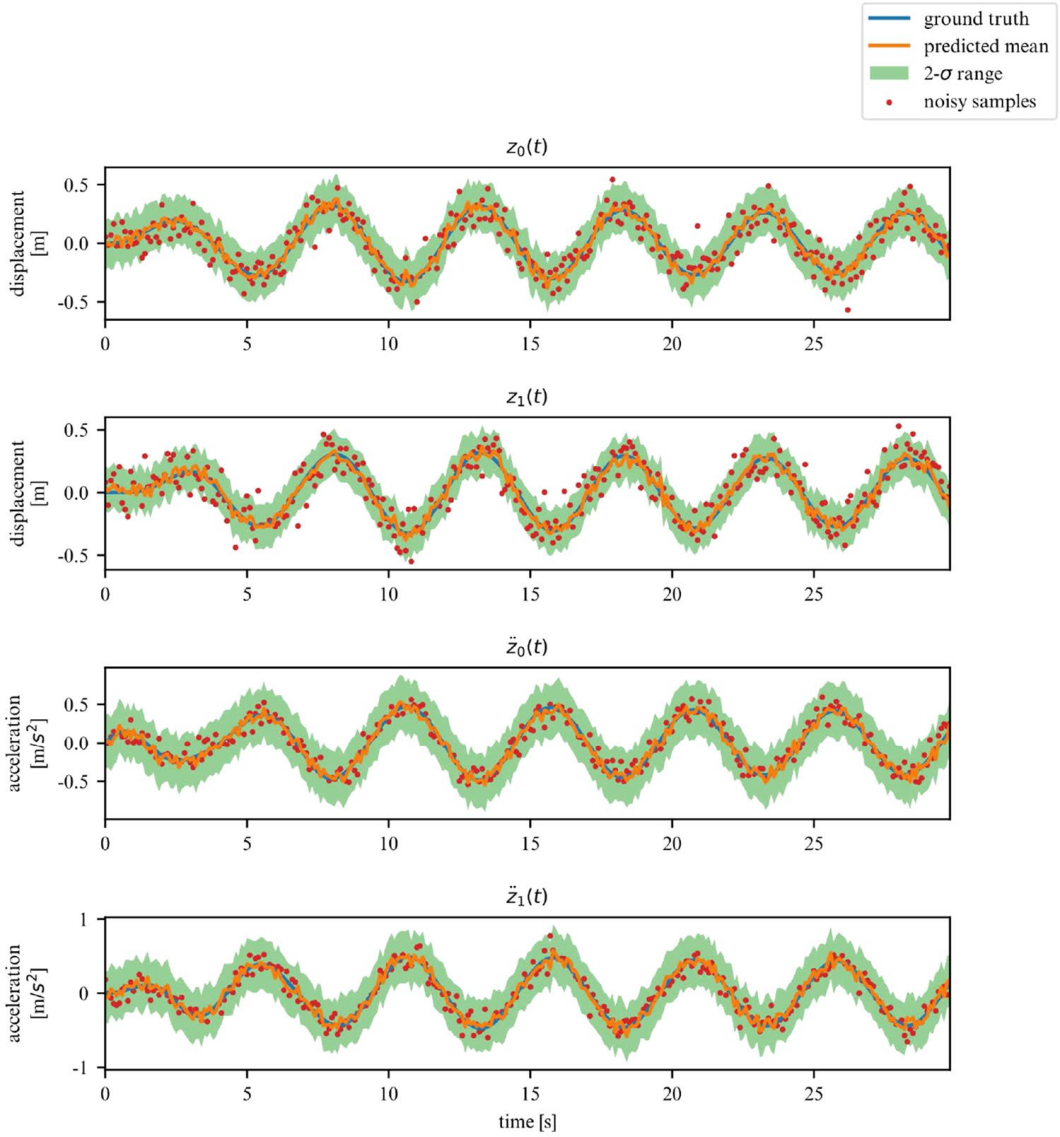
Forced dissipative 2DOF system observations for $\gamma = 2.0$.

We retrain the 2DOF duffing oscillator model with values of $\varvec{\gamma } = [0, 2]$ and $f = [0.5, 3]$. Here, the matrix $\textbf{D}$ is used to project the excitation of a subset of DOFs. For our experiments, $\textbf{D}_{1,1} = 1$ whereas the rest of $\textbf{D}$ is left to be equal to 0 so as to apply the forcing on the first DOF. We only consider the first DOF to be directly excited. As examples, we display below the predictions for a fixed $f = 1.5$ and $\gamma = 0.3, 0.9, 2.0$ on Figs. 25, 26 and 27 respectively.

### Comparison with other encoders

To compare our encoders with those of Krishnan et al.^12^, we decide to measure the Mean Squared Error (MSE) between the predicted mean and the ground truth trajectory. Furthermore, we also count the percentage of noisy datapoints that are outside the $\mu \pm 2 \sigma$ range. Each metric is computed over the test set (10% of the full dataset, i.e. 500 samples) and averaged. We implement the RNN and BiRNN encoders from the original DMM^12^, our NODE encoder and our Symplectic NODE encoder. All experiments are performed with the same random seed.

**Table:** 

**Encoder**	**RNN**		**BiRNN**		**ODE** (Ours)		**Symplectic ODE** (Ours)	
	MSE	Outlier	MSE	Outlier	MSE	Outlier	MSE	Outlier
Linear 2DOF	4.18	0.357%	4.12	0.288%	**0.73**	**0.038%**	1.56	0.056%
Linear 3DOF	1.12	0.175%	1.14	0.209%	1.02	0.244%	**0.76**	**0.031%**
Duffing 2DOF	1.69	38.954%	1.73	42.50%	1.88	2.71%	**1.01**	**0.88%**
Pendulum 2DOF	1.64	0.514%	1.59	0.316%	1.43	0.610%	**1.42**	**0.687%**
Linear 2DOF Dissipative	20.95	0.115%	15.04	0.146%	2.82	0.391%	**2.24**	**1.073%**
Duffing 2DOF Dissipative Sinusoidal Forcing	231.10	2.639%	147.13	0.654%	134.27	0.600%	**101.72**	**1.765%**

In the most simple experiment, the base NODE encoder is able to outperform its symplectic counterpart. However, for more complex dynamics, the symplectic encoder is able to make more accurate predictions than the other encoders.

## Conclusion

We presented a variation of the DMM that is able to learn a physics-informed latent representation from noisy samples of a MDOF system. In particular, the use of symplectic encoders, derived from the Hamiltonian formalism, successfully introduces the property of energy presentation to the latent space. Our model is able to learn the dynamics of a variety of linear and nonlinear system dynamics, namely linear systems, duffing oscillators and double pendulums. Our models can also be applied to the non-autonomous case. In physical terms, we are able to account for both dissipation and external forces.

Our future works in the field of dynamical system will focus on incorporating additional latent biases in our model. For example, the exact measure of the energy is still a problem to be solved. In addition, we will also explore way to scale our solutions to systems with a much larger number of DOFs.

## Data Availability

All datasets used to train and validate the models can be generated using the “simulate.py” script from https://github.com/kbacsa-ethz/phys-stoch with a desktop computer within approximately 10 min.

## List of symbols

*t*: time index variable associated to a data sample.
*T*: time horizon, i.e., maximum value of the time index variable.
$\tau$: arbitrary time index variable.
*h*: discrete integration time step constant.
*n*: number of integration steps for a forward integration scheme.
$\textbf{x}$: observable random variable.
*F*: dimension of observed random variable.
$\textbf{z}$: latent random variable.
*G*: dimension of latent random variable.
*V*: generative model for the mean latent variable.
*W*: generative model for the mean observed variable.
*S*: generative model for the standard deviation latent variable.
*R*: generative model for the standard deviation observed variable.
$\textbf{h}$: hidden layer variable of a neural network.
$\theta$: parameters of VAE decoder.
$\phi$: parameters of VAE encoder.
$\psi$: parameters for arbitrary model.
$\Psi$: number of parameters for arbitrary model.
$\beta$: parameters for model of the mean latent variable.
$\delta$: parameters for model of the standard deviation latent variable.
$\eta$: parameters for model of the mean observed variable.
$\kappa$: parameters for model of the standard deviation observed variable.
*p*: true probabilistic distribution.
*q*: approximate probabilistic distribution.
$D_{\textrm{KL}}$: Kullback-Leibner divergence.
$$\mathfrak {J}$$: loss function.
$\Phi$: function approximating the flow of an ODE.
$\mathcal {H}$: Hamiltonian of a dynamical system.
$\mathcal {L}$: Lagrangian for Lagrangian multiplier method.
$\mathfrak {L}$: continuous Lagrangian of a dynamical system.
$\mathfrak {L}^d$: discrete Lagrangian of a dynamical system.
$\textbf{q}$: standard position vector of a dynamical system.
$\textbf{p}$: standard momentum vector of a dynamical system.
$\mathbf {\alpha }$: standard angle vector of a pendulum system.
$\textbf{l}$: standard length vector of a pendulum system.
$\textbf{u}$: continuous state-space function a dynamical system.
*g*: arbitrary quadrature.
$$\lambda $$: Lagrange multiplier.
$\mathfrak {T}$: kinetic energy of a dynamic system.
$\mathfrak {U}$: potential energy of a dynamical system.
$\textbf{M}$: mass matrix of a dynamical system.
$\textbf{C}$: dissipation matrix of a dynamical system.
*K*: potential function of a dynamical system.
$\textbf{K}_l$: linear spring potential matrix of a dynamical system.
$\textbf{K}_d$: nonlinear spring potential matrix of a dynamical system.
$$\textbf{I}$$: identity matrix.
$$\textbf{0}$$: zero matrix.
*D*: forced excitation function of a dynamical system.
$\textbf{D}$: forcing matrix of a dynamical system.
$\omega _{\textrm{max}}$: function that returns the maximum eigenvalue of the input.
$\gamma$: amplitude of forced excitation of a dynamical system.
*f*: frequency of forced excitation of a dynamical system.
$$\mathcal {G}$$: gravitational constant.
$\mathcal {N}$: normal probability distribution.
$\mathcal {U}$: uniform probability distribution.
$\mu$: mean of an arbitrary distribution.
$\sigma$: standard deviation of an arbitrary distribution.
$L_E$: number of layers for the emission neural network.
$N_E$: width of layers for the emission neural network.
$N_T$: width of layers for the transmission neural network.
$L_{\textrm{RNN}}$: number of layers for the recurrent neural network in the encoder.
$L_P$: number of layers for the energy neural network.
$N_P$: width of layers for the energy neural network.
